# Development and validation of the Work–Home Integration Questionnaire (WHIQ)

**DOI:** 10.1111/apps.12456

**Published:** 2022-12-28

**Authors:** Andrea Noja, Bettina Kubicek, Nejc Plohl, Sara Tement

**Affiliations:** ^1^ Department of Psychology, Faculty of Natural Sciences University of Graz Graz Austria; ^2^ Department of Psychology, Faculty of Arts University of Maribor Maribor Slovenia

**Keywords:** burnout, scale validation, well‐being, work–family conflict, work‐home integration

## Abstract

The boundaries between work and private life are gradually blurring. More and more employees are involved in work during leisure time through cognitions, emotions or behaviours, in both negative and positive ways. This so‐called work‐home integration (WHI) can, on the one hand, hampers the necessary recovery from work and result in strain but, on the other hand, also restores resources and result in beneficial outcomes. In order to enhance our understanding of WHI and capture all its different forms, we suggest a new conceptualisation and measure of WHI. We therefore developed and validated the Work‐Home Integration Questionnaire (WHIQ) in English, German and Slovene simultaneously using two cross‐sectional studies (Study 1: *N* = 848; Study 2: *N* = 555) and a two‐wave longitudinal study with a time lag of 1 month (Study 3: *N =* 379). Confirmatory factor analysis confirmed a three‐factor structure with (1) negative cognitive‐affective involvement, (2) positive cognitive‐affective involvement and (3) behavioural involvement. Moreover, the WHIQ showed measurement invariance across the three languages and the results provide evidence for convergent, discriminant and incremental validity. Overall, the WHIQ is a reliable, valid and short measure to assess the extent to which employees are involved in work during leisure time.

## INTRODUCTION

Increasing flexibilisation and digitalisation enables employees to work anytime, anywhere. These advances in the world of work can contribute to organisational productivity and make it easier for employees to balance work and private life (Parent‐Thirion et al., 2016). However, they can also bring challenges and lead to constant availability and blurred boundaries between work and home (Kubicek & Tement, 2016). Hence, employees increasingly integrate work and home by spending their leisure time involved in work‐related cognitions and emotions (e.g. worrying about work‐related problems) or behaviours (e.g. responding to work‐related emails).

Although *work‐home integration* (WHI) and its effects on employee well‐being and health have been extensively studied in the past years (Blanco‐Encomienda et al., 2020; Cobb et al., 2022; Wendsche & Lohmann‐Haislah, 2017), there is no clear concept and no associated measure that encompasses all potential forms of WHI. Previous conceptualisations of WHI are either too broad or lack adequate precision as they focus only on some aspects of WHI. This field of research also notoriously suffers from concept proliferation (i.e. different overlapping concepts of WHI; Podsakoff et al., 2016) and ‘jingle‐jangle’ fallacies (i.e. same concepts labelled differently or different concepts labelled similarly; Allen et al., 2014; Cobb et al., 2022). The lack of conceptual clarity also impairs the operationalisation of WHI. Existing instruments either focus on one global form of WHI (e.g. Clark, 2002; Powell & Greenhaus, 2010) instead of considering multiple forms and valences, or they focus only on one specific aspect of WHI, while neglecting other aspects. They either capture work‐related cognitions (e.g. problem‐solving pondering scale; Querstret & Cropley, 2012) or behaviours (e.g. work interrupting nonwork behaviour scale; Kossek et al., 2012) during leisure time. Only a few instruments consider the affective component (e.g. affective rumination scale; Querstret & Cropley, 2012). Yet none of them includes cognitive‐affective and behavioural involvement at once. Furthermore, existing instruments primarily consider negative WHI. Positive WHI is only acknowledged in terms of cognitions (e.g. negative and positive work rumination scale; Frone, 2015) but not in terms of affect or behaviours. Although behaviours do not have a negative or positive connotation, they can be appraised as more negative or positive depending on individual perceptions (i.e. behavioural involvement valence). In sum, prior conceptualisations and the associated measures yield valuable insights, yet do not account for all different forms and valences of WHI, that is, negative cognitive‐affective involvement, positive cognitive‐affective involvement and behavioural involvement (valence).

With the aforementioned limitations of previous constructs and instruments in mind, we conceptualise, develop and validate the Work‐Home Integration Questionnaire (WHIQ). We base our conceptualisation of WHI on the most overarching and broadest definition, which is the *permeability* construct (described in more detail below). We then identify the key attributes of WHI by comparing our definition with existing concepts and instruments. Next, we develop the WHIQ based on our conceptualisation and validate the questionnaire in English, German and Slovene simultaneously using two cross‐sectional studies and one two‐wave longitudinal study. With this refined conceptualisation and comprehensive scale development, we provide insights into the complex phenomenon of WHI and advance scholarly knowledge on WHI.

A new conceptualisation and measurement of WHI will benefit research on employee stress and health in at least three ways. First, a clear conceptual definition of WHI will help to better understand the key attributes of WHI and distinguish it from other concepts, thereby reducing confusion among researchers (Podsakoff et al., 2016). In addition, a match between the conceptualisation and measurement is crucial to establish a framework for future WHI research. Second, the WHIQ can be used to identify qualitatively and quantitatively distinguishable profiles of WHI components and unravel their impact on employee health and well‐being. The multiple WHI forms may occur simultaneously (e.g. high negative cognitive‐affective involvement and high behavioural involvement), in isolation (e.g. high negative cognitive‐affective involvement but low behavioural involvement) or sequentially (e.g. negative cognitive‐affective involvement leads to behavioural involvement or vice versa) and may impact employee health differently. Individuals showing positive cognitive‐affective involvement may experience fewer health impairments than employees with negative cognitive‐affective or behavioural involvement. Thus, for a more complete picture, it is useful to consider all three forms of WHI. Third, the WHIQ can be used to uncover similarities and differences in the antecedents and outcomes of the WHI components. For example, negative cognitive‐affective involvement may have different antecedents and outcomes than positive cognitive‐affective or behavioural involvement.

### Conceptualisation of WHI

We define WHI as the frequency of being psychologically involved in the work domain while physically located in the home domain (Ashforth et al., 2000; Matthews, Barnes‐Farrell, & Bulger, 2010). For example, employees may reflect on work‐related achievements or finish a work‐related report while at home. We build our conceptualisation of WHI on the broadest and most overarching definition, which is the *permeability* construct. According to Clark (2000), permeability describes ‘the degree to which elements from other domains may enter’ (p. 756). Ashforth et al. (2000) specified that permeability means being ‘physically located in the role's domain’ while being ‘psychologically and/or behaviorally involved in another role’ (p. 474). Matthews, Barnes‐Farrell, and Bulger (2010) further suggested focusing on the frequency instead of the degree of the involvement. As WHI is based on specific, countable episodes (e.g. writing work‐related e‐mails), the construct can be captured more accurately by considering the frequency of involvement. The frequency rating allows to capture the *actual* involvement in one domain while being physically in the other domain rather than the *potential* involvement. Moreover, in contrast to existing definitions of permeability, WHI focuses on a specific direction of permeability, namely, on work entering the home domain. As previous research showed, boundary strength at work is stronger than at home (Hecht & Allen, 2009), and employees tend to integrate work into their nonwork domain more often than vice versa (Frone et al., 1992). Also, work to home integration can be more easily influenced and modified by organisations than home to work integration, which may be determined by various factors outside of the organisations' influence, such as schools, family or partners. In sum, we aim to refine the permeability construct by including the concrete form (i.e. cognitive‐affective and behavioural) of individuals' involvement (Ashforth et al., 2000; Clark, 2000), the frequency (Matthews, Barnes‐Farrell, & Bulger, 2010) and direction of the involvement from work to home.

In line with previous research, WHI is considered to be multidimensional, including cognitive‐affective and behavioural involvement (Ashforth et al., 2000; Capitano et al., 2019; Clark, 2000; Cobb et al., 2022). This conceptualisation is in line with cognitive‐behavioural approaches, which view human experience as a product of three elements—cognitions, emotions and behaviours (Scott & Dryden, 1996). These three elements are core intrapsychic processes and are referred to as the ABC of psychology. However, it does not seem feasible to investigate cognitions and emotions separately, as they are closely related (Duncan & Barrett, 2007; Storbeck & Clore, 2007). Whereas some existing approaches assume that emotions follow from cognitive appraisals (Cropley et al., 2012), others suggest that emotions arise prior to cognitive elaborations and regulate cognitive processes (Storbeck & Clore, 2007). As there is evidence for both approaches, cognitions and emotions seem to be strongly interconnected, with cognitive and affective processes tending to co‐occur within individuals. For example, when individuals worry about work, they may also feel anxious, or conversely, when individuals feel anxious about work, they may generate negative cognitions about work concurrently. Furthermore, negative cognitions *and* emotions have stronger relationships with employee health and well‐being compared with negative cognitions *without* emotions (Jimenez et al., 2021), highlighting the relevance of considering both cognitions *and* emotions jointly. Therefore, we consider cognitive‐affective involvement as one form of WHI. In addition to cognitive‐affective involvement, individuals can also be involved in work through behaviours, which are more concrete and observable. Such behaviours include finishing a report and talking about work during leisure time. Hence, on the basis of theoretical arguments and empirical findings, we suggest to differentiate two forms of WHI, cognitive‐affective and behavioural involvement. We thereby extend previous research, which mostly focused either on global forms of WHI (e.g. Clark, 2002; Powell & Greenhaus, 2010) or examined only one specific aspect of it (e.g. Fritz & Sonnentag, 2006; Frone, 2015).

In addition to the type of integration (i.e. cognitive‐affective and behavioural), WHI can be differentiated into positive or negative involvement based on its valence. Employees may be proud of something they have accomplished at work and think about the positive event at home, or they may be nervous about an upcoming work meeting while with family. Even if performing a behaviour tends to be neutral, individuals may evaluate them negatively or positively depending on individual characteristics and circumstances (Cobb et al., 2022; Lazarus & Folkman, 1984). Thus, including the frequency *and* perceived valence (i.e. negative and positive) not only of cognitions/emotions but also of behaviours (i.e. behavioural involvement valence) may provide new insights and a better understanding of WHI. Although existing constructs tend to focus on negative forms of WHI, positive forms should not be neglected, as they can provide resources and have a protective function (Binnewies et al., 2009). Moreover, negative and positive forms of WHI seem to have different antecedents (e.g. job demands and job resources) and different outcomes (e.g. well‐being and health; Jimenez et al., 2021; Wendsche et al., 2021).

### Subdimensions of WHI

WHI comprises three subdimensions: (1) negative cognitive‐affective involvement, (2) positive cognitive‐affective involvement and (3) behavioural involvement. *Negative cognitive‐affective involvement* describes work‐related negative cognitions and emotions (e.g. worry, anxiety, rumination and anger) during leisure time. This involvement occurs in a repetitive, automatic and intrusive manner, where cognitions and emotions can influence and reinforce each other (Cropley & Zijlstra, 2011). Negative cognitive‐affective involvement can refer to the future and the past, with worry and anxiety involving the anticipation of future tasks at work, and rumination and anger focusing on past events (Martin & Tesser, 1996; Shipp et al., 2009). By considering these temporal orientations, we are able to capture the varieties of cognitive‐affective involvement and thus adequately reflect how people think and feel (Shipp et al., 2009; Wendsche et al., 2021). However, we do not consider future and past orientations as distinct forms of WHI because they share similar antecedents and outcomes, and previous research failed to provide evidence for their strict separation (Gohm et al., 1996).

*Positive cognitive‐affective involvement* deals with work‐related positive cognitions and emotions (e.g. positive thoughts, enthusiasm and pride) during leisure. Similar to negative cognitive‐affective involvement, positive cognitions and emotions can also influence and reinforce each other (Cropley & Zijlstra, 2011) and can be future‐ and past‐oriented. Thinking and feeling positively about future work‐related events or incidents is described as anticipation, whereas reminiscence refers to past work events (Martin & Tesser, 1996).

*Behavioural involvement* ranges from briefly checking emails to completing an unfinished work task during leisure time. New technologies such as smartphones, laptops or the possibility of connecting to work via virtual private networks facilitate these work‐related behaviours during leisure time (e.g. Derks et al., 2014). Talking to the family/partner about work or discussing work‐related issues with colleagues during leisure time is also part of this behaviour. Unlike cognitive‐affective involvement, behavioural involvement occurs in the moment and is not anticipatory or retrospective in nature. Moreover, behaviours themselves do not have a specific negative or positive connotation. Their assessment as either negative or positive depends on individual perceptions (Cobb et al., 2022; Lazarus & Folkman, 1984) and on whether the behaviour is functional in achieving work‐related goals (Martin & Tesser, 1996). For example, talking negatively about work does not necessarily have to be negative but can be quite helpful. Therefore, in addition to the frequency of behavioural involvement, we assess the *valence* of behavioural involvement to capture how individuals perceive work‐related behaviours and examine, first, whether behaviours are perceived more negatively or positively, and second, how these behaviours are related to potential outcomes. *Behavioural involvement valence* (i.e. the interplay of frequency and valence) weights the frequency of behavioural involvement according to the individual's perceived valence of the behaviour. Thus, behavioural involvement valence lies on a continuum with frequently occurring and negatively perceived behaviours at one end and frequently occurring and positively perceived behaviours at the other end. As individuals can only rate the valence of the behaviour if they actually perform this behaviour, it is important to note that the valence (without the frequency) is not a distinct form of WHI but part of behavioural involvement valence. However, behavioural involvement (without the valence) can also be used to examine the frequency of behavioural involvement independent of the individuals' perception.

### Distinction of WHI from existing constructs

In previous literature, similar constructs to WHI have been proposed. We summarised existing scales measuring similar constructs to identify similarities/differences but also potential for improvement (see Table 1). Existing constructs refer to either one global form of WHI (e.g. permeability and actual segmentation of the work domain from the family domain; Clark, 2002; Powell & Greenhaus, 2010) or focus only on one specific form of WHI and partially overlap (Weigelt et al., 2019; Wendsche et al., 2021; for further insights into work‐related cognitions, see the meta‐anaysis by Jimenez et al., 2021).

**TABLE 1:** Summary of WHI conceptualisations and measures

#	Involvement	Source	Construct	Conceptualisation	Critique(s) of conceptualisation	Measure	Critique(s) of measure
1	General involvement	Clark (2000), Clark (2002 )	Permeability	‘… the degree to which elements from other domains may enter’ (p. 756)	No consideration of multiple forms and valences No frequency of the involvement	Permeability of Home Border Scale	One‐dimensional measure: Combines different aspects of WHI into one scale (e.g. behavioural and cognitive) No affective involvement Passive behavioural involvement (e.g. ‘receive phone calls’)
2	General involvement	Ashforth et al. (2000)	Permeability	‘… the degree to which a role allows one to be physically located in the role's domain but psychologically and/or behaviorally involved in another role’ (p. 474)	No consideration of multiple forms and valences No frequency of the involvement	No corresponding measure	‐
3	Non‐involvement	Powell and Greenhaus (2010)	Segmentation	‘… the degree of actual segmentation of their work domain from their family domain that individuals experience’ (p. 517)	No consideration of multiple forms and valences No frequency of the involvement	Actual Segmentation of the Work Domain from the Family Domain	Not sufficiently precise and too unspecific No consideration of multiple forms and valences Agreement scale
4	Non‐involvement	Etzion et al. (1998)	Psychological detachment	‘… individual's sense of being away from the work situation’ (p. 579)	No valence Not sufficiently precise (*how* individuals are detaching is missing)	Work‐Related Rumination Scale, Recovery Experience Questionnaire	No valence Not sufficiently precise and too unspecific Agreement scale
5	Cognitive‐affective involvement	Cropley and Zijlstra (2011)	Problem‐solving pondering	‘… a form of thinking that may be characterized by prolonged mental scrutiny of a particular problem or an evaluation of previous work in order to see how it can be improved, but it does not involve the emotional process that sustains arousal’ (p. 11)	No affective processes Unclear valence No frequency of the involvement	Work‐Related Rumination Scale	No affective processes Unclear valence No future/past orientation Blurring the items with potential consequences (e.g. ‘I find thinking about work during my free time helps me to be creative’)
6	Cognitive‐affective involvement	Frone (2015)	Negative work rumination	‘… preoccupation with and repetitive thoughts focused on negative work experiences that may extend beyond the workday’ (p. 5)	No affective processes No frequency of the involvement	Negative and Positive Work Rumination Scale	No affective processes No future orientation
7	Cognitive‐affective involvement	Frone (2015)	Positive work rumination	‘… preoccupation with and repetitive thoughts focused on positive work experiences that may extend beyond the workday’ (p. 6)	No affective processes No frequency of the involvement	Negative and Positive Work Rumination Scale	No affective processes No future orientation
8	Cognitive‐affective involvement	Fritz and Sonnentag (2006)	Negative work reflection	‘*…* thinking about the negative aspects of one's job and considering what one does not like about it’ (p. 937)	No affective processes No frequency of the involvement	Negative and Positive Work Reflection Scale	No affective processes No future/past orientation Agreement scale
9	Cognitive‐affective involvement	Fritz and Sonnentag (2006)	Positive work reflection	‘… considering the positive aspects of one's job and realizing what one likes about it’ (p. 938)	No affective processes No frequency of the involvement	Negative and Positive Work Reflection Scale	No affective processes No future/past orientation Agreement scale
10	Cognitive‐affective involvement	Cropley and Zijlstra (2011)	Affective rumination	‘… cognitive state characterised by the appearance of intrusive, pervasive, recurrent thoughts, about work, which are negative in affective terms’ (p. 10)	No positive counterpart No frequency of the involvement	Work‐Related Rumination Scale	No future/past orientation Blurring the items with potential consequences (e.g. ‘Do you become tense when you think about work‐related issues during your free time?’)
11	Cognitive‐affective involvement	Flaxman et al. (2012)	Work‐related worry and rumination	‘… cognitive content that focused explicitly on (work‐related) stressors or problems; a degree of repetitive and uncontrollable thinking; and a focus on potentially negative outcomes occurring in the past and/or future’ (p. 16)	No affective processes No frequency of the involvement	Work‐Related Worry and Rumination Scale	No affective processes Agreement scale
12	Behavioural involvement	Kossek et al. (2012)	Work interrupting nonwork behaviour	‘… the degree to which individuals allow interruptions from one role [work role/domain] to another [home role/domain]’ (p. 114)	Focus on interruption, the type of interruption is neglected No frequency of the involvement	Work Interrupting Nonwork Behaviours Scale	Focus on interruption, the type of interruption is neglected Agreement scale Not all aspects are measured (e.g. talking about work)
13	Behavioural involvement	Matthews, Barnes‐Farrell, and Bulger (2010)	Inter‐domain transitions	‘… the number of physical and cognitive transitions made from one domain to another’ (p. 449)	No affective involvement	Family‐to‐Work Transition Scale	Operationalisation does not match the conceptualisation No affective involvement Passive behavioural involvement (e.g. receive calls) Not all aspects are measured (e.g. talking about work)

*Note*: See also Allen et al. (2014) and Cobb et al. (2022) for an overview of permeability constructs, definitions and measures. Abbreviation: WHI, Work–Home Integration.

Global forms of WHI, such as the *permeability* construct (Clark, 2002), are too broad and combine different aspects of WHI into one single concept (e.g. behaviours: ‘I receive work‐related calls while I am at home’ and cognitions: ‘I think about work‐related concerns while I am at home’), which means that precise conclusions on differential antecedents and outcomes cannot be drawn compared with our WHI subscales. In contrast, the *actual segmentation of the work domain from the family domain* (e.g. ‘I keep work life at work’; Powell & Greenhaus, 2010), that is, non‐involvement of work during leisure time, is too unspecific, ambiguous and not sufficiently precise.

However, existing constructs that address more specific forms of WHI also have several shortcomings. *Problem‐solving pondering* is defined as thinking about and particularly finding a solution to work‐related problems (Cropley & Zijlstra, 2011). Unlike cognitive‐affective involvement, problem‐solving pondering does not involve emotional processes. Moreover, no specific valence can be assigned to problem‐solving pondering. On the one hand, it can be helpful and beneficial when solving problems and annoying if no solution is found. *Negative and positive work rumination* is defined as negative and positive thinking about work during leisure time (Binnewies et al., 2009; Frone, 2015). In contrast to problem‐solving pondering, negative and positive work rumination clearly differentiates the valence, as does negative and positive cognitive‐affective involvement, but similar to problem‐solving pondering, emotional processes are neglected. Nevertheless, there are considerable overlaps between problem‐solving pondering and negative and positive work rumination as they both refer to work‐related cognitions. Similar to negative work rumination, *affective rumination* is characterised by pervasive and intrusive cognitions, but in contrast to negative work rumination, affective rumination also involves negative affect. This combination of preservative cognitions and negative affect is also represented in the construct of *work‐related worry and rumination* (Flaxman et al., 2012). Worry and rumination are closely related processes and tend to co‐occur within individuals, where worry rather involves negative affect and rumination rather involves negative cognitions. Also, the temporal orientation of worry refers to the future and rumination to the past (Martin & Tesser, 1996). Moreover, there is no positive equivalent to affective rumination or work‐related worry and rumination, such as positive cognitive‐affective involvement. *Work interrupting nonwork behaviour* is defined as work‐related behaviours that interrupt the home domain (Kossek et al., 2012). Although this interruption has a negative connotation, it can also be perceived positively. *Family‐to‐work transition* refers to the number of transitions from one domain to another (Matthews, Barnes‐Farrell, & Bulger, 2010). However, the construct again does not consider the valence. Finally, *psychological detachment* is defined as ‘switching off’ and not thinking/feeling about or performing work during leisure time (Etzion et al., 1998; Sonnentag, 2018; Sonnentag & Fritz, 2007). Previous research has shown that psychological detachment is only related to negative work‐related rumination but not to positive work‐related rumination, indicating that psychological detachment mainly refers to disengagement from negatively valenced work experiences (Jimenez et al., 2021). Hence, psychological detachment does not capture the positive aspect of WHI. Moreover, it does not differentiate between cognitive‐affective and behavioural non‐involvement (Weigelt et al., 2019).

In addition to either being too broad or construct‐deficient operationalisations, several existing instruments also blur the WHI construct with potential consequences of WHI, for instance, ‘Do you become tense when you think about work‐related issues during your free time?’. However, employees may think about work‐related issues without becoming tense. Therefore, these types of items are difficult for participants to answer. In addition, existing instruments do not incorporate the temporal orientation (future vs. past) of cognitive‐affective involvement and thus neglect the range of how people think and feel. For behavioural involvement, existing scales also include passive behaviours (e.g. receiving phone calls; Clark, 2002). However, whether people receive calls does not necessarily indicate whether people actually answer their phone. Hence, a person's active behaviours are much more relevant. Moreover, some instruments do not match the concept. For example, the definition of the concept by Matthews, Barnes‐Farrell, and Bulger (2010) describes cognitive and behavioural involvement, but the instrument measures only behavioural involvement and neglects other forms.

In sum, similar constructs and instruments of WHI already exist, but they have some shortcomings. Our conceptualisation of WHI is an improvement because, in contrast to previous conceptualisations, it explicitly focuses on the actual involvement in work while being in the home domain. The development of the WHIQ follows a theory‐driven approach as we consider its multiple forms (i.e. cognitive‐affective and behavioural) and valences (i.e. negative and positive) instead of one global form of WHI (e.g. Clark, 2002; Powell & Greenhaus, 2010) or only one specific aspect of WHI (e.g. Fritz & Sonnentag, 2006; Frone, 2015). Moreover, previous constructs seem to suffer from construct deficiency, as they share considerable degrees of variance (Weigelt et al., 2019), yet do not capture all forms of WHI. Specifically, most constructs neglect positive cognitive‐affective involvement and behavioural involvement valence. Regarding the operationalisation, we aim to measure WHI in a more fine‐grained way. Our goal is to avoid redundancy yet be adequately precise and integrate the relevant forms of WHI in one instrument.

### Scale development and validation process

For the development and validation of the WHIQ, we followed a multistep process (Hinkin, 1998) with (1) item generation, (2) content validity and item reduction, (3) questionnaire administration (Study 1), (4) further item reduction, (5) psychometric properties, (6) nomological network and (7) replication (Studies 2 and 3).

In a first step, we developed the items in English, German and Slovene. As cultural and linguistic characteristics can restrict the development, translation and validation of questionnaires in other languages, we used a simultaneous test development approach, in which linguistic and cultural diversity can be included in the early stages of questionnaire development (Tanzer & Sim, 2013). We developed the items using a theory‐based approach and the following structure: cognitive‐affective, behavioural, negative valence, positive valence, future orientation and past orientation. When developing the items, existing scales were consulted (Binnewies et al., 2009; Capitano et al., 2019; Carlson & Frone, 2003; Cropley et al., 2012; Flaxman et al., 2012; Frone, 2015; Kossek et al., 2006, 2012; Querstret & Cropley, 2012; Sauerland, 2018; Van Katwyk et al., 2000), and items were reformulated so that they fit the proposed structure (e.g. include the temporal orientation). Items that measured other aspects or items with overly complex wording were omitted. For the forms of WHI not yet captured by existing scales, we generated new items (e.g. positive cognitive‐affective involvement; see Supporting Information for an overview of all adapted and newly generated items). Because the relationship between cognitions and emotions has not yet been clarified and it is unclear whether emotional processes arise as a consequence of or precede cognitive processes, the cognitive and emotional items were formulated separately. In general, we tried to keep the items simple and short, with consistent wording. In total, we generated an initial pool of 42 English items, which were then translated into German and Slovene by experts using a back‐translation procedure. We chose a five‐point frequency scale (1 = *[almost] never* to 5 = *[almost] always*) instead of an agreement scale to capture the actual frequency of involvement (Matthews, Barnes‐Farrell, & Bulger, 2010). Moreover, we included the response option 0 (*not applicable*). If participants indicated that they at least *rarely* perform a specific WHI behaviour, they were additionally asked to rate the valence of the behavioural involvement (−2 = *very negative* to 2 = *very positive*, 0 = *neutral*). Thus, the valence subscale may contain missing values when participants *(almost) never* perform a specific behaviour. Behavioural involvement valence was computed to combine both the frequency and the valence of the behaviour in one scale by multiplying each behavioural involvement item (9, 10, 11, 12; from which 1 was subtracted so that the response format corresponds to 0 = *never* and 4 = *always*) with the respective valence item (9a, 10a, 11a, 12a) and then calculating an overall mean (e.g. Henrich & Herschbach, 2000; Lindner et al., 2016). Participants who indicate that they *(almost) never* perform a specific WHI behaviour (and who are thus not shown the corresponding valence item) receive a value of 0. The values of behavioural involvement valence range from −8 to +8, with positive values for frequently occurring and positively perceived behaviours and negative values for frequently occurring and negatively perceived behaviours. However, the frequency of behavioural involvement without the valence can also be used to examine the main effect of behavioural involvement.

In a second step, we assessed the content validity and pretested the item pool in the three languages with the help of experts (work and clinical psychologists) and laypeople (employees). The participants (*N* = 19) were recruited from the respective universities of the authors as well as from several companies in Austria and Slovenia. We used sorting techniques, think‐aloud techniques and probing techniques (Collins, 2003) to check how the participants understood the items and how accurately they categorised them into the WHIQ subscales. On the basis of the pretests, we made minor changes and removed items that were misunderstood, unclear or not correctly assigned to the respective subdimension by at least one‐third of the participants. Thus, ambiguous items in one language version were automatically dropped in the other two versions to ensure high content validity. Finally, the preliminary pool comprised 37 items (see Supporting Information).

In a third step, we conducted a first validation study with a diverse sample of working adults. With the use of data from this first study, we refined the questionnaire and further reduced the items to avoid redundancy and improve the practical use (Step 4). We then examined the psychometric properties by testing the internal consistencies, factor structure and measurement invariance (Step 5) and assessed the nomological network of the WHIQ by examining its convergent, discriminant and incremental validity (Step 6). In a seventh step, we conducted a second validation study and replicated the results from Study 1 regarding the psychometric properties and nomological network of the finalised WHIQ to enhance generalisability. Finally, we conducted a third study using a two‐wave longitudinal study design with a time lag of 1 month to reaffirm the nomological network of the WHIQ and address concerns about common method bias. Table 2 summarises the validation steps with the respective methods and samples.

**TABLE 2:** Overview of studies, samples and steps

Study	Samples	Country	*N*
Pretest	Sample 1: Experts and laypeople: English, German, Slovene	Austria, Slovenia	19
Study 1	Sample 2: Respondi: English	United Kingdom	300
	Sample 2a/b: randomly selected half	United Kingdom	151/149
	Sample 3: Respondi: German	Germany	326
	Sample 3a/b: randomly selected half	Germany	165/161
	Sample 4: Mediana: Slovene	Slovenia	222
	Sample 4a/b: randomly selected half	Slovenia	111/111
	Sample 5: Samples 2b, 3b, 4b	United Kingdom, Germany, Slovenia	421
Study 2	Sample 6: Organisational sample: German	Austria	150
	Sample 7: Organisational sample: Slovene	Slovenia	405
	Sample 8: Samples 6, 7	Austria, Slovenia	555
Study 3	Sample 9: Prolific: English	United Kingdom	379

Steps	Methods	Samples
Item generation	Theory‐based item generation	‐
Content validity and item reduction	Sorting, think‐aloud and probing techniques	1
Further item reduction	Automated item selection	2a, 3a, 4a
Psychometric properties	Reliability, confirmatory factor analysis, metric invariance	2b, 3b, 4b, 6, 7, 9
Nomological network	Correlations and hierarchical multiple regression	5, 8, 9

### Theoretical background and hypotheses

On the basis of the theoretical foundation, we assume that the WHIQ consists of three subdimensions: (1) negative cognitive‐affective involvement, (2) positive cognitive‐affective involvement and (3) behavioural involvement.

Hypothesis 1 The WHIQ represents a three‐factor structure.

#### Convergent validity: Relations to other WHI factors

To test convergent validity, we examined whether the subdimensions of the WHIQ are associated with similar constructs. For negative cognitive‐affective involvement, we considered problem‐solving pondering, affective rumination and negative work rumination. These three concepts all relate to some aspect of negative cognitive‐affective involvement (Cropley & Zijlstra, 2011; Frone, 2015). Therefore, we assume positive relations of problem‐solving pondering, affective rumination and negative work rumination with negative cognitive‐affective involvement.

Hypothesis 2 WHI through negative cognitive‐affective involvement is positively related to (a) problem‐solving pondering, (b) affective work rumination and (c) negative work rumination.

For positive cognitive‐affective involvement, we considered problem‐solving pondering and positive work rumination (i.e. repetitive thinking about positive work experiences; Frone, 2015). Although both concepts neglect positive affective involvement, both refer to positive cognitive involvement at least to some extent. Thus, we assume positive relationships of problem‐solving pondering and positive work rumination with positive cognitive‐affective involvement:

Hypothesis 3 WHI through positive cognitive‐affective involvement is positively related to (a) problem‐solving pondering and (b) positive work rumination.

For behavioural involvement, we considered work interrupting nonwork behaviour (Kossek et al., 2012) and family‐to‐work transitions (Matthews, Barnes‐Farrell, & Bulger, 2010) as related measures. Although Kossek et al.'s measure emphasises interruptions rather than the type of behaviour, both work interrupting nonwork behaviour and behavioural involvement refer to performing work‐related activities during leisure time. Family‐to‐work transitions mainly refer to physical transitions from the work domain to the family domain (Matthews, Barnes‐Farrell, & Bulger, 2010) and are therefore also related to behavioural involvement. Thus, we propose:

Hypothesis 4 WHI through behavioural involvement is positively related to (a) work interrupting nonwork behaviour and (b) family‐to‐work transitions.

#### Discriminant validity: Relation to personality factors

With regard to discriminant validity, we assume that personality factors are related to, but still different from, the WHIQ. We chose neuroticism, extraversion and conscientiousness as personality factors, as they show the closest relationships with WHI (Michel et al., 2011). Individuals high in neuroticism exhibit anxiety, nervousness and worry. Because these experiences overlap with negative cognitive‐affective involvement, we expect neuroticism to be positively related to the negative cognitive‐affective subscale. In line with this argument, neuroticism was shown to be negatively related to psychological detachment (Wendsche & Lohmann‐Haislah, 2017) and positively to problem‐solving pondering and affective rumination (Hamesch et al., 2014). Positive cognitive‐affective and behavioural involvement should only be weakly associated with neuroticism.

Conscientious individuals tend to be disciplined, organised and have a high level of control over their cognitions, emotions and behaviours, especially the negative ones (Michel et al., 2011). These individuals consequently engage more strongly in the domain they are physically located in (Michel et al., 2011). Thus, the three WHI subscales should be negatively associated with conscientiousness.

Extraversion indicates the tendency to be a social, active and dominant individual with high positive emotionality. In the work context, individuals high in extraversion are more likely to seek proactive solutions and successfully handle job demands, suggesting an association with WHI. Previous research has shown that extraversion has a negative relationship with negative WHI and a positive relationship with positive WHI (Michel et al., 2011). Thus, we propose:

Hypothesis 5 WHI through negative cognitive‐affective involvement is positively related to (a) neuroticism and negatively related to (b) conscientiousness and (c) extraversion.

Hypothesis 6 WHI through positive cognitive‐affective involvement is negatively related to (a) conscientiousness and positively related to (b) extraversion.

Hypothesis 7 WHI through behavioural involvement is negatively related to (a) conscientiousness and positively related to (b) extraversion.

#### Incremental validity: Relations to work, family and individual outcomes

To test incremental validity, we assessed whether the WHIQ adds to existing measures when it comes to the prediction of various outcomes. As outcomes, work‐related (i.e. burnout and work engagement) and general well‐being (i.e. well‐being, negative mood and positive mood), work‐to‐family conflict (WFC) and work‐to‐family enrichment (WFE) were considered.

One indicator of work‐related well‐being is burnout with its two dimensions emotional exhaustion and disengagement/cynicism. Emotional exhaustion is characterised by a feeling of being overwhelmed and fatigued by work, whereas disengagement and cynicism refer to distancing oneself from the job (Demerouti & Nachreiner, 1998). Negative cognitive‐affective involvement may result in both exhaustion and disengagement/cynicism. With regard to exhaustion, we assume that negative cognitive‐affective involvement keeps the stress from work cognitively and emotionally active and prevents the necessary recovery during off‐job time. If recovery from work is not possible over a longer period of time, this may deplete employees emotional and physical resources (Hobfoll, 1989) and eventually manifest in exhaustion in the long run. In addition, negative cognitive‐affective involvement may also result in disengagement/cynicism, as employees may protect themselves from prolonged stress activation by developing a cynical attitude towards work (Demerouti & Nachreiner, 1998). Insufficient recovery from the job may affect not only burnout but also general well‐being and mood. General well‐being comprises the subjective well‐being of individuals and (negative and positive) affect or mood (Diener, 1984; Topp et al., 2015). Previous studies suggested that insufficient recovery during off‐job time and the resulting depletion of resources due to negative cognitive‐affective involvement is associated with lower general well‐being (Firoozabadi et al., 2018; Kinnunen et al., 2017). Finally, negative cognitive‐affective involvement may also increase WFC, which refers to the incompatibility of work and family roles, where engagement in the work role deteriorates the functioning in the family role (Carlson et al., 2000). Negative cognitive‐affective involvement may lead to insufficient involvement in the family role as individuals may contribute less time or energy to the family role and increase WFC (Junker et al., 2020). Thus, we propose:

Hypothesis 8 WHI through negative cognitive‐affective involvement is negatively related to (a) work‐related well‐being and (b) general well‐being and positively related to (c) WFC over and above similar scales.

Positive cognitive‐affective involvement seems to be associated with lower levels of burnout and higher levels of work engagement, general well‐being and WFE (Fritz & Sonnentag, 2006; Junker et al., 2020; Weigelt et al., 2019). It might have a protective function, replenish resources (Binnewies et al., 2009) and reduce exhaustion. In addition, positive cognitive‐affective involvement may decrease negative attitudes towards work and thus decrease disengagement/cynicism. Positive cognitive‐affective involvement may lead to a positive (re)appraisal of work experiences and a more positive perspective for the following work day, thus increasing work engagement that is defined as a ‘positive, fulfilling, work‐related state of mind that is characterised by vigour, dedication, and absorption’ (Bakker & Demerouti, 2008, p. 209). Furthermore, positive cognitive‐affective involvement may not only positively affect work‐related well‐being but also increase general well‐being and positive mood. Finally, positive cognitive‐affective involvement may also affect the work‐family interface, namely, WFE, which occurs when participating in the work role promotes the functioning in the family role (Carlson et al., 2006). Accordingly, work‐related positive cognitive‐affective involvement may extend to the family role and increase WFE (Junker et al., 2020). Thus, we propose:

Hypothesis 9 WHI through positive cognitive‐affective involvement is positively related to (a) work‐related well‐being, (b) general well‐being and (c) WFE over and above similar scales.

Behavioural involvement reduces the time and energy that can be invested in other domains and therefore prevents employees from recovering from work during off‐job time, which, in turn, may lead to impaired well‐being (e.g. Derks et al., 2014; Matthews, Barnes‐Farrell, & Bulger, 2010). However, the individual valence of behavioural involvement has, to the best of our knowledge, not yet been studied. As the valence affects potential outcomes, we use the interplay of frequency and valence to best predict outcomes. However, we also include the direct effect of the frequency of behavioural involvement to investigate the additional variance explained by behavioural involvement valence. We assume that frequently occurring and positively perceived behavioural involvement has a protective function and replenishes resources (similar to positive cognitive‐affective involvement). Thus, it shows negative relationships with impaired work‐related well‐being and WFC and positive relationships with general well‐being and WFE.

Hypothesis 10 WHI through positive behavioural involvement is positively related to (a) work‐related well‐being, (b) general well‐being and (c) WFE and negatively related to (d) WFC over and above similar scales.

## STUDY 1

### Method

#### Samples

Three independent samples were recruited for Study 1. The English and German samples were gathered by the online panel *Respondi* (ISO‐certified: ISO 26362), and the Slovene sample was gathered by the research institute *Mediana*. The following inclusion criteria were used: employees, at least 18 years old, not spending most of their working time outside of the workplace (e.g. telework) and working at least 30 h per week.

A total of 848 participants fully completed the survey in Study 1. The age of the participants ranged from 18 to 66 years (*M* = 42.43, *SD* = 11.35), and there was a balanced gender ratio (49% female). Half of the participants had at least a bachelor's degree. On average, participants worked 33 h per week (*SD* = 12.61) and were employed by their organisation for 11 years (*SD* = 9.91). About one‐fourth (26%) of the participants worked in shifts, and about one‐third (32%) had a leading position.

#### Measures

All measures that were not available in the respective language version were translated by experts using the back‐translation procedure.

**WHI** was assessed with 37 items from the pretested item pool.

**Other WHI factors:** *Problem‐solving pondering* (e.g. ‘After work I tend to think of how I can improve my work‐related performance’) was assessed using the 5‐item subscale from the Work‐Related Rumination Questionnaire (WRRQ; English: Querstret & Cropley, 2012; German: Lang & Kraus, 2012). The responses were given on a 5‐point scale (1 *= never/very seldom* to 5 = *very often/always*).

*Affective rumination* (e.g. ‘Do you become tense when you think about work‐related issues during your free time?’) was measured using the 5‐item subscale from the WRRQ (response format: 1 *= never/very seldom* to 5 = *very often/always*).

*Work interrupting nonwork behaviour* (e.g. ‘I allow work to interrupt me when I spend time with my family or friends’) was measured using the 5‐item subscale from the Boundary Management Scale (Kossek et al., 2012). The response format ranged from 1 (*disagree strongly*) to 5 (*agree strongly*).

**Personality factors:** *Neuroticism* (e.g. ‘I am someone who worries a lot’), *conscientiousness* (e.g. ‘I am someone who is fascinated by art, music, or literature’) and *extraversion* (e.g. ‘I am someone who is dominant, acts as a leader’) were measured using the extra‐short form of the Big Five Inventory (BFI‐2‐XS; English: Soto & John, 2017; German: Rammstedt et al., 2020). The responses were given on a 5‐point scale (1 = *disagree strongly* to 5 = *agree strongly*).

**Outcomes:** *Burnout* was measured with the 16‐item Oldenburg Burnout Inventory (English: Demerouti et al., 2003; German: Demerouti & Nachreiner, 1998; Slovene: Sedlar et al., 2015). The responses to the two subscales, emotional exhaustion (e.g. ‘There are days that I feel already tired before I go to work’) and disengagement (e.g. ‘It happens more and more often that I talk about my work in a derogatory way’), were given on a 5‐point scale (1 = *disagree strongly* to 5 = *agree strongly*).

*Work–family conflict* (WFC) was measured using the 6‐item Work–Family Conflict Scale (English: Carlson et al., 2000; German: Wolff & Höge, 2011; Slovene: Tement et al., 2010). Responses to the items (e.g. ‘My work keeps me from my family activities more than I would like’) were given on a 5‐point scale (1 = *disagree strongly* to 5 = *agree strongly*).

*Work*–*family enrichment* (WFE) was measured using the 6‐item Work–Family Enrichment Scale (English: Carlson et al., 2006; Slovene: Tement et al., 2010). Responses to the items (e.g. ‘My involvement in my work helps me to understand different viewpoints and this helps me be a better family member’) were given on a 5‐point scale (1 = *disagree strongly* to 5 = *agree strongly*).

#### Statistical analyses

We used a cross‐validation approach and divided the overall sample into two randomly selected subsamples (see Table 2). Based on the first subsample, items were reduced. The psychometric properties and the nomological network were calculated using the second subsample.

### Results

#### Item selection

To refine the questionnaire and keep it as simple and short as possible, the number of items was reduced. An automated item selection procedure in analogy to the stuart‐package from R was used to find an optimal item combination in all languages (Schultze, 2018; Schultze & Eid, 2018). Based on a brute‐force approach, confirmatory factor analyses were conducted with all possible combinations of items in all three languages until the best item combination in terms of model fit was found. We assessed the model fit, using the chi‐square (χ^2^), the comparative fit index (CFI), the Tucker–Lewis index (TLI), the root mean square error of approximation (RMSEA) and the standardised root mean square residuals (SRMRs) with their respective cut‐off values (CFI and TLI ≥ .950, RMSEA ≤ .060 and SRMR ≤ .080 for good model fit; and CFI and TLI ≥ .900, RMSEA and SRMR ≤ .080 for acceptable model fit; Bentler, 1992; Hu & Bentler, 1999; Vandenberg & Lance, 2000). Because of the non‐normal distribution of the items, the maximum likelihood estimator and the corrected version were used (Satorra‐Bentler). The selection of the final WHIQ items was based not only on the output of the automated item selection but also on the content of the items. We tried to keep the variation of the items as high as possible and included different facets of WHI to avoid redundancy and high similarity of the items. Overall, the initial 37‐item questionnaire was shortened to 12 items with acceptable to excellent model fit statistics for the three‐factor model (English: χ_SB_^2^ = 51.88, *df* = 51, CFI = .998, TLI = .997, RMSEA = .015, SRMR = .063; German: χ_SB_^2^ = 80.69, *df* = 51, CFI = .940, TLI = .923, RMSEA = .070, SRMR = .068; Slovene: χ_SB_^2^ = 61.94, *df* = 51, CFI = .972, TLI = .964, RMSEA = .051, SRMR = .066).

#### Psychometric properties

Cronbach's alphas were calculated to determine the internal consistencies of the subscales. For each WHIQ subscale, Cronbach's alpha was above the limit of .70 (see Table 5; Nunnally & Bernstein, 1994). The other study variables also had acceptable Cronbach's alphas, except for the Big Five subscales (i.e. extraversion and conscientiousness) measured by the extra‐short version of the Big Five Inventory, which showed lower values. However, previous studies have found similar reliability coefficients for the extra‐short version of the Big Five Inventory and demonstrated strong retest reliability, structural validity and predictive power (Rammstedt et al., 2020; Soto & John, 2017).

Using the lavaan‐package from R (Rosseel, 2012), a confirmatory factor analysis was conducted on the second half of the sample, which supported a three‐factor structure with (1) negative cognitive‐affective involvement, (2) positive cognitive‐affective involvement and (3) behavioural involvement (see Table 3). Compared with the best‐fitting two‐factor model (combining positive cognitive‐affective involvement and behavioural involvement) and a one‐factor model, the three‐factor model yielded a significantly better model fit, supporting Hypothesis 1.

**TABLE 3:** Confirmatory factor analysis and invariance test of the WHIQ in Study 1

Model	Sample	χ_SB_^2^	*df*	*p*	CFI	TLI	RMSEA [90% CI]	SRMR	AIC	Model comparison	Δχ_SB_^2^	Δ*df*	*p*	ΔCFI	ΔRMSEA
M1: 3‐factor model	a) English	51.75	51	<.001	.998	.998	.013 [.000–.072]	.062	3702.70						
	b) German	86.66	51	<.001	.928	.907	.076 [.047–.103]	.069	4262.75						
	c) Slovene	61.94	51	<.001	.972	.964	.051 [.000–.092]	.066	3300.49						
M2: 2‐factor model	a) English	101.37	53	<.001	.881	.852	.108 [.076–.140]	.102	3779.09	M2a‐M1a	49.62	2	<.001	−.117	.095
	b) German	135.60	53	<.001	.829	.787	.115 [.091–.139]	.095	4316.96	M2b‐M1b	48.94	2	<.001	−.099	.039
	c) Slovene	91.13	53	<.001	.906	.883	.093 [.059–.125]	.076	3332.27	M2c‐M1c	29.19	2	<.001	−.066	.042
M3: 1‐factor model	a) English	229.69	54	<.001	.575	.481	.202 [.176–.230]	.153	3965.28	M3a‐M1a	177.94	3	<.001	−.423	.189
	b) German	182.70	54	<.001	.731	.672	.142 [.120–.165]	.108	4370.06	M3b‐M1b	96.04	3	<.001	−.197	.066
	c) Slovene	182.62	54	<.001	.689	.620	.167 [.141–.194]	.130	3441.85	M3c‐M1c	120.68	3	<.001	−.283	.116
**Invariance test**															
M1i: Configural model		196.16	153	<.001	.967	.958	.055 [.028–.076]	.061	11,337.94						
M2i: Factor loadings constrained		220.20	171	<.001	.963	.957	.055 [.030–.075]	.071	11,328.81	M2i‐M1i	24.04	18	.150	−.004	.000
M3i: Factor loadings, variances and covariances constrained		235.14	183	<.001	.961	.958	.054 [.031–.074]	.088	11,322.06	M3i‐M1i	38.98	30	.125	−.006	−.001

*Note*: *N =* 421 (*n*_*English*_ = 149, *n*_*German*_ = 161, *n*_*Slovene*_ = 111). 2‐factor model: (1) negative cognitive‐affective involvement, (2) positive cognitive‐affective involvement and behavioural involvement.

Abbreviations: AIC, Akaike information criterion; CFI, comparative fit index; RMSEA, root mean square error of approximation; SRMR, standardised root mean square residual; TLI, Tucker–Lewis index; WHIQ, Work–Home Integration Questionnaire.

Finally, measurement invariance was assessed among the English, German and Slovene versions. The configural model without cross‐group constraints was compared with a fully constrained model with factor loadings, factor variances and factor covariances constrained across the samples. The results revealed a non‐significant chi‐square difference test, a low ΔCFI (≤ .010) and a low ΔRMSEA (≤ .015), confirming metric invariance across the three languages (Vandenberg & Lance, 2000).

The items and standardised factor loadings are presented in Table 4. The factor loadings of the behavioural subscale are slightly lower than those of the other subscales due to the higher content variation of the items but still acceptable (Hinkin, 1998).

**TABLE 4:** Items, means (*M*), standard deviations (*SD*) and standardised factor loadings of the WHIQ for the three languages in Study 1

No.	Subscale	Sub‐group	Items			Factor loadings		
			*During my leisure time …*	*M*	*SD*	English	German	Slovene
1	*Negative cognitive‐affective involvement*	Future	… I worried about how I would deal with upcoming work tasks or issues.	2.48	1.07	.79	.78	.68
2		Past	… I ruminated about things that went wrong at work.	2.53	1.10	.86	.86	.82
3		Future	… I felt anxious because of upcoming work meetings or tasks.	2.32	1.09	.82	.69	.90
4		Past	… I felt angry because of things that had happened at work.	2.58	1.08	.73	.69	.84
5	*Positive cognitive‐affective involvement*	Future	… I thought positively about upcoming work tasks.	2.63	1.02	.68	.66	.56
6		Past	… I reflected on the things that went well at work.	2.72	0.97	.85	.76	.64
7		Future	… I felt enthusiastic because of upcoming work tasks.	2.47	1.07	.82	.70	.74
8		Past	… I felt proud because of my work‐related achievements.	2.98	1.06	.66	.62	.77
9	*Behavioural involvement*	‐	… I responded to work‐related phone calls or e‐mails.	2.40	1.26	.61	.63	.62
9a	*Valence*	‐	Please rate the impact that this behaviour has on your leisure time.	−0.27	0.84	‐	‐	‐
10	*Behavioural involvement*	‐	… I studied work‐related materials.	2.03	1.16	.75	.74	.67
10a	*Valence*	‐	Please rate the impact that this behaviour has on your leisure time.	0.02	0.95	‐	‐	‐
11	*Behavioural involvement*	‐	… I talked about work‐related things with my family or friends.	2.64	1.14	.65	.59	.69
11a	*Valence*	‐	Please rate the impact that this behaviour has on your leisure time.	0.08	0.85	‐	‐	‐
12	*Behavioural involvement*	‐	… I organized work‐related things with my colleagues or clients.	1.71	0.98	.80	.49	.73
12a	*Valence*	‐	Please rate the impact that this behaviour has on your leisure time.	−0.15	1.04	‐	‐	‐

*Note*: *N =* 421 (*n*_*English*_ = 149, *n*_*German*_ = 161, *n*_*Slovene*_ = 111).

Abbreviation: WHIQ, Work–Home Integration Questionnaire.

**TABLE 5:** Intercorrelations and Cronbach's alpha of study variables in Study 1

		*M*	*SD*	1	2	3	4	5	6	7	8	9	10	11	12	13	14	15
1	Negative cognitive‐affective involvement	2.48	0.91	(.86)														
2	Positive cognitive‐affective involvement	2.70	0.80	.34***	(.78)													
3	Behavioural involvement	2.23	0.88	.43***	.44***	(.75)												
4	Valence	−0.04	0.73	−.24***	.27***	.00	‐											
5	Behavioural involvement valence^a^	−0.03	1.16	−.25***	.26***	.06	.82***	‐										
6	Problem‐solving pondering	2.47	0.84	.62***	.47***	.54***	−.05	−.02	(.88)									
7	Affective rumination	2.42	1.06	.71***	.03	.39***	−.39***	−.37***	.60***	(.94)								
8	Work interrupting nonwork behaviour	2.02	1.01	.45***	.28***	.62***	−.07	−.05	.55***	.43***	(.89)							
9	Neuroticism	2.63	0.89	.57***	−.03	.17***	−.30***	−.27***	.33***	.56***	.22***	(.70)						
10	Conscientiousness	3.83	0.77	−.35***	−.11*	−.18***	−.06	−.06	−.27***	−.26***	−.17***	−.45***	(.60)					
11	Extraversion	3.03	0.75	−.21***	.21***	.18***	.20***	.24***	.01	−.17***	.06	−.36***	.26***	(.49)				
12	Exhaustion	2.41	0.52	.53***	−.21***	.07	−.38***	−.38***	.25***	.62***	.20***	.51***	−.24***	−.31***	(.83)			
13	Disengagement	2.36	0.53	.33***	−.34***	−.02	−.38***	−.33***	.02	.40***	.09	.34***	−.17***	−.24***	.65***	(.78)		
14	WFC	2.90	1.01	.50***	−.02	.24***	−.34***	−.28***	.31***	.58***	.35***	.42***	−.17***	−.16**	.67***	.42***	(.92)	
15	WFE	3.21	0.93	−.13**	.40***	.15***	.37***	.37***	.08	−.23***	.08	−.23***	.07	.26***	−.41***	−.47***	−.22***	(.93)

*Note*: *N* = 421 (except for valence *N* = 389).

Abbreviations: WFC, work–family conflict; WFE, work–family enrichment.

^a^ High values: frequently occurring and positively perceived behaviour; low values: frequently occurring and negatively perceived behaviour.

* *p* < .05.

** *p* < .01.

*** *p* < .001.

#### Nomological network

Convergent and discriminant validity were tested using bivariate correlations (see Table 5).

##### Convergent validity: Relations to other WHI factors

Negative cognitive‐affective involvement showed significant high correlations with problem‐solving pondering (*r* = .62) and affective rumination (*r* = .71), supporting Hypotheses 2a and b. Positive cognitive‐affective involvement showed a significant moderate correlation with problem‐solving pondering (*r* = .47), supporting Hypothesis 3a. Behavioural involvement showed a significant high correlation with work interrupting nonwork behaviour (*r* = .62), supporting Hypothesis 4a.

##### Discriminant validity: Relations to personality factors

Negative cognitive‐affective involvement showed positive correlations with neuroticism (*r* = .57) and negative correlations with conscientiousness (*r* = −.35) and extraversion (*r* = −.21), supporting Hypotheses 5a–c. Positive cognitive‐affective involvement indicated a negative relationship with conscientiousness (*r* = −.11) and a positive relationship with extraversion (*r* = .21), supporting Hypotheses 6a and b. Behavioural involvement showed a negative correlation with conscientiousness (*r* = −.18) and a positive correlation with extraversion (*r* = .18), supporting Hypotheses 7a and b. However, we also found a positive relationship between behavioural involvement and neuroticism (*r* = .17).

##### Incremental validity: Relations to outcomes

Incremental validity was tested using multiple regression analyses. Exhaustion, disengagement, WFC and WFE were considered as outcomes. For these analyses, behavioural involvement valence was used to account for the negative/positive valence. Behavioural involvement (without the valence) was also included in the regressions to examine the additional variance explained by behavioural involvement valence (see Table 6).

**TABLE 6:** Incremental validity of the WHIQ on exhaustion, disengagement, WFC and WFE in Study 1

	Exhaustion		Disengagement		WFC		WFE	
Problem‐solving pondering	−.19***	−.28***	−.34***	−.43**	−.06	−.13*	.34***	.36***
Affective rumination	.73***	.57***	.60***	.46**	.61***	.49***	−.43***	−.39***
Negative cognitive‐affective involvement		.30***		.27***		.24***		−.08
*R* ^2^	.40***	.44***	.23***	.27***	.34***	.36***	.13***	.13***
Δ*R* ^2^		.04***		.04***		.02***		.00
Problem‐solving pondering	.25***	.44***	.02	.23***	.31***	.41***	.08	−.14**
Positive cognitive‐affective involvement		−.42***		−.45***		−.22***		.47***
*R* ^2^	.06***	.20***	.00	.16***	.10***	.13***	.01	.18***
Δ*R* ^2^		.14***		.16***		.03***		.17***
Work interrupting nonwork behaviour	.20***	.19**	.09	.12*	.35***	.29***	.08	.02
Behavioural involvement		−.02		−.08		.08		−.12*
Behavioural involvement valence^a^		−.37***		−.32***		−.27***		.36***
*R* ^2^	.04***	.18***	.01	.12***	.12***	.19***	.01	.15***
Δ*R* ^2^		.14***		.11***		.07***		.14***

*Note*: *N* = 421. Standardised betas are reported.

Abbreviations: WFC, work–family conflict; WFE, work–family enrichment; WHIQ, Work–Home Integration Questionnaire.

^a^ High values: frequently occurring and positively perceived behaviour; low values: frequently occurring and negatively perceived behaviour.

* *p* < .05.

** *p* < .01.

*** *p* < .001.

As expected, negative cognitive‐affective involvement was positively related to exhaustion (β = .30), disengagement (β = .27) and WFC (β = .24) over and above problem‐solving pondering and affective rumination, supporting Hypotheses 8a and c. Positive cognitive‐affective involvement was negatively related to exhaustion (β = −.42), disengagement (β = −.45) and positively related to WFE (β = .47) over and above problem‐solving pondering, supporting Hypotheses 9a and c. Behavioural involvement valence (high values indicate frequently occurring and positively perceived behaviours) was negatively related to exhaustion (β = −.37), disengagement (β = −.32), WFC (β = −.27) and positively related to WFE (β = .36) over and above work interrupting nonwork behaviour and behavioural involvement, supporting Hypotheses 10a, c and d.

### Discussion Study 1

In Study 1, we developed and validated the WHIQ in English, German and Slovene to measure the involvement in work during leisure time. Results confirmed a three‐factor structure including (1) negative cognitive‐affective involvement, (2) positive cognitive‐affective involvement and (3) behavioural involvement and indicated metric invariance across the three language versions. Moreover, the WHIQ subscales were associated with similar measures (i.e. problem‐solving pondering, affective rumination and work interrupting nonwork behaviour) and differed from personality factors, supporting convergent and discriminant validity. The findings regarding incremental validity indicated that the WHIQ subscales are the strongest predictor of burnout and WFE over and above existing scales and uniquely contribute to the prediction of WFC.

Despite promising results, Study 1 has some limitations that warrant further study. First, the data were collected via online panels, which have some disadvantages (e.g. panel bias). However, the online panels used, *Respondi* and *Mediana*, ensure representative samples by regularly recruiting new participants, keeping the participation frequency low and intrinsic motivation high due to low incentives for participation. Nevertheless, the replication of the results is required to enhance generalisability.

Second, Study 1 was conducted with the 37‐item version of the WHIQ, and it was shortened based on the data collected. A further study is needed to confirm and replicate the three‐factor structure of the final 12‐item version of the questionnaire (Hinkin, 1998).

Third, for testing convergent validity of the WHIQ in Study 1, we used established instruments for measuring work‐related rumination and behaviours (i.e. problem‐solving pondering, affective rumination and work interrupting nonwork behaviour). However, assessing relations with similar constructs that focus on the separation between negative and positive WHI is also relevant to demonstrate adequate validity.

## STUDY 2

In Study 2, we aimed to retest the three‐factor structure in the final 12‐item version of the WHIQ and replicate the results of the psychometric properties and nomological network from Study 1 using an organisational sample.

### Method

#### Samples

Two independent samples were recruited for Study 2, with a total of 555 employees fully completing the survey. The Austrian sample (*n* = 150) was recruited in several organisations from different fields of work (e.g. health care, real estate and sales), and the Slovene sample (*n* = 405) was recruited in several universities. The age of the participants ranged from 19 to 68 years (*M* = 41.30, *SD* = 10.69), and the majority of the participants were female (68%). Due to the data collected at universities, the sample was highly educated (75% with at least a master's degree). On average, participants worked 44 h per week (*SD* = 9.73) and were employed by their organisation for 12 years (*SD* = 9.52). About one‐fourth of the participants (27%) had a leading position in their company.

#### Measures

**WHI** was measured using the 12‐item scale developed in Study 1.

**Other WHI factors.** *Negative and positive work rumination* were assessed using the 8‐item scale from the Negative and Positive Work Rumination Scale (Frone, 2015). In contrast to Study 1, we particularly focus on the distinction between negative and positive WHI in Study 2. Therefore, we used negative (e.g. ‘How often do you replay negative work events in your mind even after you leave work?’) and positive work rumination (e.g. ‘How often do you find yourself preoccupied with positive aspects of your job even after you leave work?’) to assess convergent validity. Responses to the two subscales were given on a 5‐point scale (1 *= never/very seldom* to 5 = *very often/always*).

*Work interrupting nonwork behaviour* was measured with the same scale as in Study 1.

**Outcome.** *Burnout* was measured with two subscales of the Maslach Burnout Inventory‐General Scale (MBI‐GS; Maslach et al., 1996) to use a different conceptualisation and measure of burnout than in Study 1. The responses to the two subscales, emotional exhaustion (e.g. ‘I feel emotionally drained from my work’) and cynicism (e.g. ‘I have become less interested in my work since I started this job’), with a total of 10 items, were given on a 7‐point scale (1 = *never* to 7 = *every day*).

### Results

#### Psychometric properties

Cronbach's alphas of all study variables were above .70 (see Table 7).

**TABLE 7:** Intercorrelations and Cronbach's alpha of study variables in Study 2

		*M*	*SD*	1	2	3	4	5	6	7	8	9	10
1	Negative cognitive‐affective involvement	2.83	0.95	(.88)									
2	Positive cognitive‐affective involvement	2.95	0.79	−.04	(.83)								
3	Behavioural involvement	3.00	0.98	.49***	.26***	(.79)							
4	Valence	−0.17	0.77	−.45***	.30***	−.17***	‐						
5	Behavioural involvement valence^a^	−0.45	1.85	−.45***	.28***	−.24***	.87***	‐					
6	Negative work rumination	3.30	0.92	.68***	−.03	.47***	−.35***	−.36***	(.94)				
7	Positive work rumination	2.99	0.79	.14***	.57***	.36***	.15***	.16***	.32***	(.88)			
8	Work interrupting nonwork behaviour	2.80	1.19	.47***	.17***	.80***	−.21***	−.26***	.48***	.35***	(.91)		
9	Exhaustion	4.15	1.26	.59***	−.19***	.34***	−.42***	−.39***	.51***	.03	.41***	(.92)	
10	Cynicism	3.30	1.62	.52***	−.37***	.18***	−.47***	−.44***	.46***	−.17***	.25***	.80***	(.88)

*Note*: *N* = 555 (except for valence *N* = 550).

^a^ High values: frequently occurring and positively perceived behaviour; low values: frequently occurring and negatively perceived behaviour.

* *p* < .05.

** *p* < .01.

*** *p* < .001.

The results of the confirmatory factor analysis showed acceptable model fit and confirmed the three‐factor structure with (1) negative cognitive‐affective involvement, (2) positive cognitive‐affective involvement and (3) behavioural involvement, supporting Hypothesis 1 (Austria: χ_SB_^2^ = 112.51, *df* = 51, CFI = .910, TLI = .884, RMSEA = .093, SRMR = .087; Slovenia: χ_SB_^2^ = 169.36, *df* = 51, CFI = .942, TLI = .925, RMSEA = .080, SRMR = .065). The three‐factor model yielded a significantly better model fit compared with the best‐fitting two‐factor model (combining negative cognitive‐affective involvement and behavioural involvement; Austria: Δχ_SB_^2^ = 93.46, Δ*df* = 2, *p* < .001, ΔCFI = −.136, ΔRMSEA = .052; Slovenia: Δχ_SB_^2^ = 293.35, Δ*df* = 2, *p* < .001, ΔCFI = −.148, ΔRMSEA = .069) and a one‐factor model (Austria: Δχ_SB_^2^ = 205.14, Δ*df* = 3, *p* < .001, ΔCFI = −.358, ΔRMSEA = .109; Slovenia: Δχ_SB_^2^ = 784.14, Δ*df* = 3, *p* < .001, ΔCFI = −.389, ΔRMSEA = .137). When assessing measurement invariance among the two samples, the configural model was compared with a model with constrained factor loadings. The results revealed a significant chi‐square difference test, a somewhat high ΔCFI (.014) but low ΔRMSEA (.005). Hence, the next step was to check for partial metric invariance. After freeing two items (‘I felt anxious because of upcoming work meetings or tasks.’, ‘I reflected on the things that went well at work.’), we found support for partial metric invariance (ΔCFI = .007; ΔRMSEA = .001; see Supporting Information).

#### Nomological network

##### Convergent validity: Relations to other WHI factors

Negative cognitive‐affective involvement showed a significant positive correlation with negative work rumination (*r* = .68), supporting Hypothesis 2c. Positive cognitive‐affective involvement showed a significant positive correlation with positive work rumination (*r* = .57), supporting Hypothesis 3b. Behavioural involvement showed a significant positive correlation with work interrupting nonwork behaviour (*r* = .80), supporting Hypothesis 4a (see Table 7 for Pearson's correlations of all study variables).

##### Incremental validity: Relation to burnout

As expected, negative cognitive‐affective involvement was positively related to exhaustion (β = .46) and cynicism (β = .39) over and above negative work rumination, supporting Hypothesis 8a. Positive cognitive‐affective involvement was negatively related to exhaustion (β = −.31) and cynicism (β = −.41) over and above positive work rumination, supporting Hypothesis 9a. Behavioural involvement valence (high values indicate frequently occurring and positively perceived behaviours) was negatively related to exhaustion (β = −.31) and cynicism (β = −.40) over and above work interrupting nonwork behaviour and behavioural involvement, supporting Hypothesis 10a (see Table 8).

**TABLE 8:** Incremental validity of the WHIQ on exhaustion and cynicism in Study 2

	Exhaustion		Cynicism	
Negative work rumination	.51***	.20***	.46***	.19***
Negative cognitive‐affective involvement		.46***		.39***
*R* ^2^	.26***	.37***	.21***	.29***
Δ*R* ^2^		.11***		.08***
Positive work rumination	.03	.21***	−.17***	.06
Positive cognitive‐affective involvement		−.31***		−.41***
*R* ^2^	.00	.07***	.03***	.14***
Δ*R* ^2^		.07***		.11***
Work interrupting nonwork behaviour	.41***	.32***	.25***	.23***
Behavioural involvement		.01		−.10
Behavioural involvement valence^a^		−.31***		−.40***
*R* ^2^	.17***	.26***	.06***	.22***
Δ*R* ^2^		.09***		.16***

*Note*: *N* = 555. Standardised betas are reported.

Abbreviation: WHIQ, Work–Home Integration Questionnaire.

^a^ High values: frequently occurring and positively perceived behaviour; low values: frequently occurring and negatively perceived behaviour.

* *p* < .05.

** *p* < .01.

*** *p* < .001.

### Discussion Study 2

In Study 2, we confirmed the three‐factor structure of the WHIQ and replicated the results of Study 1 in Austrian and Slovene organisational samples. We again tested for measurement invariance, but the results revealed that the factor loadings were only partially invariant across the Austrian and Slovene samples. This could be due to differences in sample sizes and sample characteristics such as education and occupation (Byrne et al., 1989). However, measurement invariance did not hold for only two items and is thus negligible. As in Study 1, the results provide evidence for convergent and incremental validity. Overall, we replicated the results of Study 1.

## STUDY 3

Study 3 used a two‐wave study design with a time lag of 1 month to retest the three‐factor structure of the WHIQ and to further establish the nomological network and address concerns about common method bias. We decided on a 4‐week interval as previous research on related constructs used a similar time frame (i.e. between 2 and 5 weeks; Sonnentag & Fritz, 2015).

### Method

#### Sample

Data for Study 3 were gathered via the online panel *Prolific Academic* in the United Kingdom. A total of 493 employees fully completed the survey at T1. Of these, 42 were screened out as the quality of the data was insufficient. Thus, 451 employees got an invitation for T2, and 408 answered the survey at T2 (response rate: 90%). After screening qualitatively insufficient data, the final sample consisted of 379 employees.

The age of the participants ranged from 20 to 64 years (*M* = 37.24, *SD* = 9.88), and there was a balanced gender ratio (47% female). Half of the participants had a higher secondary education (45%), and about one‐fourth had a lower (28%) or higher (27%) education. On average, employees worked 40 h per week (*SD* = 5.47). About 15% worked in shifts, and 42% had a leading position. On average, participants were employed by their organisation for 7 years (*SD* = 6.71).

#### Measures

We assessed all of the following measures at both T1 and T2. In addition, participants were instructed to think about the past 4 weeks when answering the items.

**WHI** was measured using the scale developed in Study 1.

**Other WHI factors.** *Negative and positive work rumination* were measured with the same scale as in Study 2.

*Family‐to‐work transitions* (e.g. ‘How often have you received calls from co‐workers or your supervisor while at home?’) were assessed using the 5‐item subscale from the Inter‐Domain Transitions Scale (Matthews, Barnes‐Farrell, & Bulger, 2010). The responses were given on a 6‐point scale (1 *= at no time* to 6 = *all of the time*).

**Outcomes.** *Burnout* was measured with the same scale as in Study 2.

*Work‐engagement* (e.g. ‘I am enthusiastic about my job’) was assessed using the 3‐item ultra‐short version of the Utrecht Work Engagement Scale (response format: 1 = *never* to 7 = *always*; Schaufeli et al., 2019).

*General well‐being* was measured with the WHO‐5 Well‐Being Index (Topp et al., 2015). Responses to the items (e.g. ‘Over the past 4 weeks I have felt cheerful and in good spirits’) were given on a 6‐point scale (1 *= at no time* to 6 = *all of the time*).

*Negative and positive mood* were each measured with 3 items from the Mood Scale (Abele‐Brehm & Brehm, 1986). Participants indicated on a 5‐point scale (1 = *[almost] never* to 5 = *[almost] always*) how often they felt a negative mood (i.e. strained, irritated and grumpy) or a positive mood (i.e. serene, relaxed and calm) in the last 4 weeks.

*WFC* was measured using the 3‐item Work–Family Conflict Scale (Matthews, Kath, & Barnes‐Farrell, 2010). Responses to the items (e.g. ‘I had to miss family activities due to the amount of time I had to spend on work responsibilities’) were given on a 5‐point scale (1 = *strongly disagree* to 5 = *strongly agree*).

*WFE* was measured using the 3‐item version of the Work‐Family Enrichment Scale (Kacmar et al., 2014). Responses to the items (e.g. ‘My involvement in my work helped me to understand different viewpoints and this helped me be a better family member’) were given on a 5‐point scale (1 = *strongly disagree* to 5 = *strongly agree*).

### Results

#### Psychometric properties

Cronbach's alphas of all study variables were above the standard cutoff of .70, except for WFC, with a slightly lower value of .68 (see Table 9). However, the reliability of WFC is close to the limit of .70, and previous research indicated strong reliability as well as construct and content validity of the scale (Matthews, Kath, & Barnes‐Farrell, 2010).

**TABLE 9:** Intercorrelations and Cronbach's alpha of study variables in Study 3

		*M*	*SD*	1	2	3	4	5	6	7	8	9	10	11	12	13	14	15	16
1	T1 Negative cognitive‐affective involvement	2.55	0.92	(.87)															
2	T1 Positive cognitive‐affective involvement	2.75	0.89	.01	(.91)														
3	T1 Behavioural involvement	2.13	0.77	.48***	.27***	(.76)													
4	T1 Valence	−0.16	0.55	−.28***	.28***	−.17**	‐												
5	T1 Behavioural involvement valence^a^	−0.27	0.99	−.34***	.24***	−.31***	.83***	‐											
6	T1 Negative work rumination	2.44	0.82	.73***	−.05	.47***	−.27***	−.31***	(.94)										
7	T1 Positive work rumination	2.12	0.71	.06	.66***	.36***	.23***	.18***	.17***	(.91)									
8	T1 Family‐to‐work transitions	1.77	0.85	.41***	.14**	.63***	−.31***	−.45***	.39***	.22***	(.81)								
9	T2 Exhaustion	3.95	1.37	.52***	−.27***	.24***	−.29***	−.31***	.49***	−.09	.30***	(.94)							
10	T2 Cynicism	3.77	1.49	.29***	−.53***	−.03	−.28***	−.28***	.27***	−.33***	.13*	.60***	(.92)						
11	T2 Work engagement	4.25	1.14	−.12*	.65***	.18***	.23***	.20***	−.08	.45***	.04	−.41***	−.73***	(.87)					
12	T2 Well‐being	3.57	1.03	−.38***	.44***	−.09	.25***	.23***	−.36***	.25***	−.12*	−.62***	−.54***	.60***	(.91)				
13	T2 Negative mood	2.76	0.86	.52***	−.18***	.27***	−.28***	−.28***	.47***	.00	.32***	.71***	.53***	−.35***	−.66***	(.87)			
14	T2 Positive mood	3.02	0.79	−.44***	.28***	−.24***	.24***	.25***	−.42***	.11*	−.24***	−.54***	−.40***	.40***	.72***	−.63***	(.84)		
15	T2 WFC	2.60	0.92	.54***	−.05	.41***	−.32***	−.34***	.48***	.06	.48***	.68***	.37***	−.19***	−.38***	.56***	−.43***	(.68)	
16	T2 WFE	3.26	0.91	−.12*	.50***	.13**	.32***	.29***	−.06	.36***	−.05	−.30***	−.52***	.57***	.39***	−.24***	.29***	−.20***	(.86)

*Note*: *N* = 379 (except for valence *N* = 360).

Abbreviations: WFC, work–family conflict; WFE, work–family enrichment.

^a^ High values: frequently occurring and positively perceived behaviour; low values: frequently occurring and negatively perceived behaviour.

* *p* < .05.

** *p* < .01.

*** *p* < .001.

The results of the confirmatory factor analysis showed acceptable model fit statistics and confirmed the three‐factor structure with (1) negative cognitive‐affective involvement, (2) positive cognitive‐affective involvement and (3) behavioural involvement, supporting Hypothesis 1 (χ_SB_^2^ = 121.97, *df* = 51, CFI = .969, TLI = .959, RMSEA = .063, SRMR = .060). The three‐factor model yielded a significantly better model fit compared with the best‐fitting two‐factor model (Δχ_SB_^2^ = 225.40, Δ*df* = 2, *p* < .001, ΔCFI = −.101, ΔRMSEA = .065) and a one‐factor model (Δχ_SB_^2^ = 1202.31, Δ*df* = 3, *p* < .001, ΔCFI = −.538, ΔRMSEA = .199; see Supporting Information).

#### Nomological network

##### _Convergent validity: Relations to other WHI factors_

Negative cognitive‐affective involvement showed a significant high positive correlation with negative work rumination (*r* = .73), supporting Hypothesis 2c. Positive cognitive‐affective involvement showed a significant high positive correlation with positive work rumination (*r* = .66), supporting Hypothesis 3b. Behavioural involvement showed a significant high positive correlation with family‐to‐work transitions (*r* = .63), supporting Hypothesis 4b (see Table 9 for Pearson's correlations of all study variables).

##### Incremental validity: Relation to outcomes

As expected, negative cognitive‐affective involvement was positively related to exhaustion (β = .34), cynicism (β = .20), negative mood (β = .39) and WFC (β = .40) and negatively related to general well‐being (β = −.25) beyond negative work rumination, supporting Hypotheses 8a–c. Positive cognitive‐affective involvement was negatively related to exhaustion (β = −.37) and cynicism (β = −.55) and positively related to work engagement (β = .62), general well‐being (β = .48), positive mood (β = .36) and WFE (β = .47) beyond positive work rumination, supporting Hypotheses 9a–c. Behavioural involvement valence (high values indicate frequently occurring and positively perceived behaviours) was negatively related to exhaustion (β = −.22), cynicism (β = −.29), negative mood (β = −.17) and WFC (β = −.15) and positively related to work engagement (β = .28), general well‐being (β = .22), positive mood (β = .17) and WFE (β = .35), beyond family‐to‐work transitions and behavioural involvement, supporting Hypotheses 10a–d (see Table 10). However, the results also showed unexpected findings that are not part of our hypotheses, which may be due to a suppression effect (e.g. the positive relationship between positive work rumination and exhaustion when positive cognitive‐affective involvement is also in the model; β = .15).

**TABLE 10:** Incremental validity of the WHIQ in Study 3

	T2 Exhaustion		T2 Cynicism		T2 Work engagement		T2 General well‐being		T2 Negative mood		T2 Positive mood		T2 WFC		T2 WFE	
T1 Negative work rumination	.49***	.25***	.27***	.13	−.08	.01	−.36***	−.17*	.47***	.18**	−.42***	−.20**	.48***	.20**	−.06	.07
T1 Negative cognitive‐affective involvement		.34***		.20**		−.13		−.25***		.39***		−.30***		.40***		−.18*
*R* ^2^	.24***	.29***	.08***	.09***	.01	.02	.13***	.16***	.22***	.29***	.17***	.21***	.24***	.31***	.00	.02*
Δ*R* ^2^		.05***		.01**		.01		.03***		.07***		.04***		.07***		.02*
T1 Positive work rumination	−.09	.15*	−.33***	.03	.45***	.04	.25***	−.07	.00	.20**	.11*	−.12	.06	.17*	.36***	.06
T1 Positive cognitive‐affective involvement		−.37***		−.55***		.62***		.48***		−.31***		.36***		−.16*		.47***
*R* ^2^	.01	.09***	.11***	.28***	.20***	.42***	.06***	.19***	.00	.06***	.01*	.09***	.00	.02*	.13***	.26***
Δ*R* ^2^		.08***		.17***		.22***		.13***		.06***		.08***		.02*		.13***
T1 Family‐to‐work transitions	.30***	.16*	.13*	.12	.04	.00	−.13*	−.02	.32***	.18**	−.24***	−.09	.48***	.30***	−.05	−.07
T1 Behavioural involvement		.07		−.19**		.26***		.00		.11		−.13*		.18**		.29***
T1 Behavioural involvement valence^a^		−.22***		−.29***		.28***		.22***		−.17**		.17**		−.15**		.35***
*R* ^2^	.09***	.13***	.02*	.10***	.00	.10***	.02*	.05***	.11***	.14***	.06***	.09***	.23***	.27***	.00	.15***
Δ*R* ^2^		.04***		.08***		.10***		.03***		.03**		.03**		.04***		.15***

*Note*: *N* = 379. Standardised betas are reported.

Abbreviations: WFC, work–family conflict; WFE, work–family enrichment; WHIQ, Work–Home Integration Questionnaire.

^a^ High values: frequently occurring and positively perceived behaviour; low values: frequently occurring and negatively perceived behaviour.

* *p* < .05.

** *p* < .01.

*** *p* < .001.

### Discussion Study 3

In Study 3, we confirmed the three‐factor structure of the WHIQ and replicated the results of Studies 1 and 2 regarding convergent and incremental validity. The findings indicate that the WHIQ subscales are associated with similar measures (i.e. negative work rumination, positive work rumination and family‐to‐work transitions) and uniquely contribute to the prediction of various outcomes (i.e. burnout, work engagement, general well‐being, negative and positive mood, WFC and WFE) over and above existing scales. We found that all results related to incremental validity hold across a time lag of 1 month. As the two‐wave study design allowed us to separate the measurement of predictors and outcomes, common method bias concerns can be at least partially ruled out.

## GENERAL DISCUSSION

The purpose of this research was to present a conceptualisation of and measure for assessing the involvement in work during leisure time. We proposed a conceptualisation of WHI that is not too general and sufficiently precise by including all theoretically grounded forms of WHI (i.e. negative cognitive‐affective involvement, positive cognitive‐affective involvement and behavioural involvement). On the basis of this conceptualisation, we developed and validated the WHIQ in English, German and Slovene following a comprehensive scale development process to better understand WHI and its relationships to established organisational constructs. Results of two cross‐sectional studies and one two‐wave longitudinal study confirmed the three‐factor structure and demonstrated good psychometric properties as well as good convergent, discriminant and incremental validity of the WHIQ.

Although earlier research assumed that cognitions and emotions are fundamentally different phenomena, modern psychological science reveals that these processes interact and are interdependent (e.g. Duncan & Barrett, 2007; Storbeck & Clore, 2007). The findings of the present research also indicate that negative and positive cognitive‐affective involvement predict outcomes above and beyond scales that capture only cognitive involvement (i.e. problem‐solving pondering and negative and positive work rumination). These findings support the assumption that a joint consideration of cognitions *and* emotions better reflects the integration of work into private life.

As behavioural involvement does not have a specific negative or positive connotation, we additionally assess the valence of behavioural involvement to capture whether behaviours are perceived more negatively or positively. We then combined the valence with the frequency of behavioural involvement using an interaction. Similar to other studies, the frequency of behavioural involvement was multiplied with a rating of the valence of behavioural involvement (e.g. Henrich & Herschbach, 2000; Lindner et al., 2016). The results indicate that behavioural involvement valence is more strongly related to several outcomes than behavioural involvement without the valence, emphasising the importance of including employees' perception of behaviours. The differing behaviour ratings among individuals, and within individuals, indicate that behaviours are not innately negative or positive. Behaviours can be helpful in achieving important goals in one domain (e.g. work) but, on the other hand, hinder the achievement of goals in the other domain (e.g. family), which may explain the different valences of behaviours. Future research should investigate what specifically contributes to negative or positive valence. However, behavioural involvement can also be used without the valence if researchers are only interested in investigating the frequency of behavioural involvement.

### Contribution of the WHIQ

The WHIQ provides valuable contributions to existing research. The conceptualisation of WHI developed in this research helps to understand the key attributes of WHI, distinguishes WHI from other concepts and facilitates the measurement. The development of the WHIQ followed a theory‐driven approach and considered the multidimensional structure of WHI, instead of focusing on one global form of WHI or only one specific aspect of WHI while neglecting other aspects. Thus, future research can use the WHIQ to examine negative cognitive‐affective involvement, positive cognitive‐affective involvement and behavioural involvement (valence) to better understand the WHI components. Specifically, the WHIQ can be used to reveal different antecedents and outcomes that provide insights into when the different forms of WHI occur in particular and which form is particularly relevant for the prediction of which outcomes.

The comprehensive scale development process of the WHIQ also contributes to the application and further development of the instrument. We simultaneously developed the questionnaire in English, German and Slovene to include linguistic characteristics in the early stages of questionnaire development and ensure cross‐cultural equivalence (Tanzer & Sim, 2013). Thus, the translation and validation of the WHIQ in further languages should be facilitated. In addition, we tried to keep the WHIQ as short and simple as possible to enhance its practical use and keep the demands for the participants low. Furthermore, the WHIQ is flexible in terms of adaptations to the family context. A home‐to‐work integration questionnaire can be developed with minor changes in wording without compromising the actual content (e.g. ‘During my work time, I studied documents related to my private life, e.g., children's teachers' evaluation’).

### Limitations and future research

The present research has certain limitations and offers directions for future research. The first limitation refers to the content of the items. The WHIQ mainly focuses on knowledge work. Especially for the behavioural subscale, some items (e.g. responding to work‐related phone calls or e‐mails) may not be suitable for certain occupational groups (e.g. assembly line or construction workers), although other items of behavioural involvement (e.g. talking with family or friends about work‐related issues) as well as the negative and positive cognitive‐affective subscales fit for all occupational groups. Future research should examine whether negative and positive cognitive‐affective involvement are more relevant for certain occupational groups, as some items from behavioural involvement may not be applicable. Although it is useful to investigate all three WHI forms, future studies can also use single subscales (e.g. only negative cognitive‐affective involvement) if researchers are interested in specific aspects of WHI.

Second, for a broader theoretical understanding of WHI, further studies are needed to better place WHI within the nomological network of established organisational constructs. Future research should investigate different antecedents and determine why WHI arises. Both workplace characteristics (e.g. time pressure, cognitive and emotional demands and job resources; Wendsche & Lohmann‐Haislah, 2017) and person‐related characteristics (e.g. maladaptive thinking patterns; Tement et al., 2020) can lead to WHI. Future research needs to examine these relationships in more detail. Moreover, different outcomes should be investigated more closely, for example, how WHI relates to objective health (via impaired/improved recovery) or to performance at work and at home.

Third, the results refer to between‐person variations and do not consider within‐person variations or cross‐level interactions. Future research may conduct diary studies and examine daily variations of the WHI subscales, for example, how the valence of behavioural involvement varies from day to day and how daily factors influence this varying valence. Future studies may also investigate negative loops of the WHI subscales, for example, if negative cognitive‐affective involvement predicts negatively perceived behavioural involvement.

### Conclusion

In this research, we suggest a new conceptualisation and measure of the involvement in work during leisure time. The WHIQ provides a reliable and valid measure in three languages (i.e. English, German and Slovene) that captures negative and positive cognitive‐affective and behavioural integration of work into the home domain. Overall, the WHIQ helps researchers expand scientific understanding of the complex relationship between WHI and employee well‐being.

## CONFLICT OF INTEREST

The authors have no conflicts of interest to declare that are relevant to the content of this article.

## ETHICS APPROVAL

All procedures performed in studies involving human participants were in accordance with the ethical standards of the institutional research committee and with the 1964 Helsinki declaration and its later amendments or comparable ethical standards.

## Supporting information


**Table S1.** The English Work‐Home Integration Questionnaire (WHIQ)
**Table S2.** The German Work‐Home Integration Questionnaire (WHIQ)
**Table S3.** The Slovene Work‐Home Integration Questionnaire (WHIQ)
**Table S4.** Questionnaire Development of the Work‐Home Integration Questionnaire (WHIQ)
**Table S5.** Confirmatory Factor Analysis and Invariance Test of the WHIQ in Study 2
**Table S6.** Confirmatory Factor Analysis of the WHIQ in Study 3 (T1)

## Data Availability

The data that support the findings of this research are available from the corresponding author upon reasonable request.

## References

[apps12456-bib-0001] Abele‐Brehm, A. , & Brehm, W. (1986). Zur Konzeptualisierung und Messung von Befindlichkeit: Die Entwicklung der Befindlichkeitsskalen (BFS). [The development of the Mood Survey.]. Diagnostica, 32(3), 209–228.

[apps12456-bib-0002] Allen, T. D. , Cho, E. , & Meier, L. L. (2014). Work–family boundary dynamics. Annual Review of Organizational Psychology and Organizational Behavior, 1(1), 99–121. 10.1146/annurev-orgpsych-031413-091330

[apps12456-bib-0003] Ashforth, B. E. , Kreiner, G. E. , & Fugate, M. (2000). All in a day's work: Boundaries and micro role transitions. Academy of Management Review, 25(3), 472–491. 10.2307/259305

[apps12456-bib-0004] Bakker, A. B. , & Demerouti, E. (2008). Towards a model of work engagement. Career Development International, 13(3), 209–223. 10.1108/13620430810870476

[apps12456-bib-0005] Bentler, P. M. (1992). On the fit of models to covariances and methodology to the Bulletin. Psychological Bulletin, 112(3), 400–404. 10.1037/0033-2909.112.3.400 1438635

[apps12456-bib-0006] Binnewies, C. , Sonnentag, S. , & Mojza, E. J. (2009). Feeling recovered and thinking about the good sides of one's work. Journal of Occupational Health Psychology, 14(3), 243–256. 10.1037/a0014933 19586220

[apps12456-bib-0007] Blanco‐Encomienda, F. J. , García‐Cantero, R. , & Latorre‐Medina, M. J. (2020). Association between work‐related rumination, work environment and employee well‐being: A meta‐analytic study of main and moderator effects. Social Indicators Research, 150(3), 887–910. 10.1007/s11205-020-02356-1

[apps12456-bib-0008] Byrne, B. M. , Shavelson, R. J. , & Muthén, B. (1989). Testing for the equivalence of factor covariance and mean structures: The issue of partial measurement invariance. Psychological Bulletin, 105(3), 456–466. 10.1037/0033-2909.105.3.456

[apps12456-bib-0009] Capitano, J. , McAlpine, K. L. , & Greenhaus, J. H. (2019). Organizational influences on work–home boundary permeability: A multidimensional perspective. In M. R. Buckley , A. R. Wheeler , J. E. Baur , & J. R. B. Halbesleben (Eds.). Research in Personnel and Human Resources Management (Vol. 37, pp. 133–172). Emerald Publishing Limited. 10.1108/S0742-730120190000037005

[apps12456-bib-0010] Carlson, D. S. , & Frone, M. R. (2003). Relation of behavioral and psychological involvement to a new four‐factor conceptualization of work‐family interference. Journal of Business and Psychology, 17(4), 515–535. 10.1023/A:1023404302295

[apps12456-bib-0011] Carlson, D. S. , Kacmar, K. M. , Wayne, J. H. , & Grzywacz, J. G. (2006). Measuring the positive side of the work–family interface: Development and validation of a work–family enrichment scale. Journal of Vocational Behavior, 68(1), 131–164. 10.1016/j.jvb.2005.02.002

[apps12456-bib-0012] Carlson, D. S. , Kacmar, K. M. , & Williams, L. J. (2000). Construction and initial validation of a multidimensional measure of work–family conflict. Journal of Vocational Behavior, 56(2), 249–276. 10.1006/jvbe.1999.1713

[apps12456-bib-0013] Clark, S. C. (2000). Work/family border theory: A new theory of work/family balance. Human Relations, 53(6), 747–770. 10.1177/0018726700536001

[apps12456-bib-0014] Clark, S. C. (2002). Communicating across the work/home border. Community, Work & Family, 5(1), 23–48. 10.1080/13668800020006802

[apps12456-bib-0015] Cobb, H. R. , Murphy, L. D. , Thomas, C. L. , Katz, I. M. , & Rudolph, C. W. (2022). Measuring boundaries and borders: A taxonomy of work‐nonwork boundary management scales. Journal of Vocational Behavior, 137, 103760. 10.1016/j.jvb.2022.103760

[apps12456-bib-0016] Collins, D. (2003). Pretesting survey instruments: An overview of cognitive methods. Quality of Life Research, 12(3), 229–238. 10.1023/A:1023254226592 12769135

[apps12456-bib-0017] Cropley, M. , Michalianou, G. , Pravettoni, G. , & Millward, L. J. (2012). The relation of post‐work ruminative thinking with eating behaviour. Stress and Health, 28(1), 23–30. 10.1002/smi.1397 22259155

[apps12456-bib-0018] Cropley, M. , & Zijlstra, F. R. H. (2011). Work and rumination. In J. Langan‐Fox & C. L. Cooper (Eds.), Handbook of stress in the occupations (pp. 487–502). 10.4337/9780857931153.00061

[apps12456-bib-0019] Demerouti, E. , Bakker, A. , Vardakou, I. , & Kantas, A. (2003). The convergent validity of two burnout instruments: A multitrait‐multimethod analysis. European Journal of Psychological Assessment, 19(1), 12–23. 10.1027//1015-5759.19.1.12

[apps12456-bib-0020] Demerouti, E. , & Nachreiner, F. (1998). Zur Spezifität von Burnout für Dienstleistungsberufe: Fakt oder Artefakt [The specificity of burnout for human services: Fact or artefact]. Zeitschrift Für Arbeitswissenschaft, 52, 82–89.

[apps12456-bib-0021] Derks, D. , van Mierlo, H. , & Schmitz, E. B. (2014). A diary study on work‐related smartphone use, psychological detachment and exhaustion: Examining the role of the perceived segmentation norm. Journal of Occupational Health Psychology, 19(1), 74–84. 10.1037/a0035076 24447222

[apps12456-bib-0022] Diener, E. (1984). Subjective well‐being. Psychological Bulletin, 95(3), 542–575. 10.1037/0033-2909.95.3.542 6399758

[apps12456-bib-0023] Duncan, S. , & Barrett, L. F. (2007). Affect is a form of cognition: A neurobiological analysis. Cognition & Emotion, 21(6), 1184–1211. 10.1080/02699930701437931 18509504 PMC2396787

[apps12456-bib-0081] Etzion, D. , Eden, D. , & Lapidot, Y. (1998). Relief from job stressors and burnout: Reserve service as a respite. Journal of Applied Psychology, 83(4), 577–585. 9729927 10.1037/0021-9010.83.4.577

[apps12456-bib-0024] Firoozabadi, A. , Uitdewilligen, S. , & Zijlstra, F. R. H. (2018). Solving problems or seeing troubles? A day‐level study on the consequences of thinking about work on recovery and well‐being, and the moderating role of self‐regulation. European Journal of Work and Organizational Psychology, 27(5), 629–641. 10.1080/1359432X.2018.1505720

[apps12456-bib-0025] Flaxman, P. E. , Ménard, J. , Bond, F. W. , & Kinman, G. (2012). Academics' experiences of a respite from work: Effects of self‐critical perfectionism and perseverative cognition on postrespite well‐being. Journal of Applied Psychology, 97(4), 854–865. 10.1037/a0028055 22545621

[apps12456-bib-0026] Fritz, C. , & Sonnentag, S. (2006). Recovery, well‐being, and performance‐related outcomes: The role of workload and vacation experiences. Journal of Applied Psychology, 91(4), 936–945. 10.1037/0021-9010.91.4.936 16834516

[apps12456-bib-0027] Frone, M. R. (2015). Relations of negative and positive work experiences to employee alcohol use: Testing the intervening role of negative and positive work rumination. Journal of Occupational Health Psychology, 20(2), 148–160. 10.1037/a0038375 25528689 PMC4372465

[apps12456-bib-0028] Frone, M. R. , Russell, M. , & Cooper, M. L. (1992). Prevalence of work‐family conflict: Are work and family boundaries asymmetrically permeable? Journal of Organizational Behavior, 13(7), 723–729. 10.1002/job.4030130708

[apps12456-bib-0029] Gohm, C. L. , Isbell, L. M. , & Wyer, R. S. (1996). Some thoughts about thinking. In R. S. Wyer (Ed.). Ruminative Thoughts: Advances in Social Cognition (pp. 81–96). Psychology Press.

[apps12456-bib-0030] Hamesch, U. , Cropley, M. , & Lang, J. (2014). Emotional versus cognitive rumination: Are they differentially affecting long‐term psychological health? The impact of stressors and personality in dental students. Stress and Health, 30(3), 222–231. 10.1002/smi.2602 25100273

[apps12456-bib-0031] Hecht, T. D. , & Allen, N. J. (2009). A longitudinal examination of the work‐nonwork boundary strength construct. Journal of Organizational Behavior, 30(7), 839–862. 10.1002/job.579

[apps12456-bib-0032] Henrich, G. , & Herschbach, P. (2000). Questions on Life Satisfaction (FLZM): A short questionnaire for assessing subjective quality of life. European Journal of Psychological Assessment, 16(3), 150–159. 10.1027//1015-5759.16.3.150

[apps12456-bib-0033] Hinkin, T. R. (1998). A brief tutorial on the development of measures for use in survey questionnaires. Organizational Research Methods, 1(1), 104–121. 10.1177/109442819800100106

[apps12456-bib-0034] Hobfoll, S. E. (1989). Conservation of resources: A new attempt at conceptualizing stress. American Psychologist, 44(3), 513–524. 10.1037/0003-066X.44.3.513 2648906

[apps12456-bib-0035] Hu, L. , & Bentler, P. M. (1999). Cutoff criteria for fit indexes in covariance structure analysis: Conventional criteria versus new alternatives. Structural Equation Modeling: A Multidisciplinary Journal, 6(1), 1–55. 10.1080/10705519909540118

[apps12456-bib-0036] Jimenez, W. P. , Hu, X. , & Xu, X. V. (2021). Thinking about thinking about work: A meta‐analysis of off‐job positive and negative work‐related thoughts. Journal of Business and Psychology, 37(2), 237–262. 10.1007/s10869-021-09742-7

[apps12456-bib-0037] Junker, N. M. , Baumeister, R. F. , Straub, K. , & Greenhaus, J. H. (2020). When forgetting what happened at work matters: The role of affective rumination, problem‐solving pondering, and self‐control in work–family conflict and enrichment. Journal of Applied Psychology, 106(11), 1750–1766. 10.1037/apl0000847 33090861

[apps12456-bib-0038] Kacmar, K. M. , Crawford, W. S. , Carlson, D. S. , Ferguson, M. , & Whitten, D. (2014). A short and valid measure of work‐family enrichment. Journal of Occupational Health Psychology, 19(1), 32–45. 10.1037/a0035123 24447219

[apps12456-bib-0039] Kinnunen, U. , Feldt, T. , Sianoja, M. , de Bloom, J. , Korpela, K. , & Geurts, S. (2017). Identifying long‐term patterns of work‐related rumination: Associations with job demands and well‐being outcomes. European Journal of Work and Organizational Psychology, 26(4), 514–526. 10.1080/1359432X.2017.1314265

[apps12456-bib-0040] Kossek, E. E. , Lautsch, B. A. , & Eaton, S. C. (2006). Telecommuting, control, and boundary management: Correlates of policy use and practice, job control, and work–family effectiveness. Journal of Vocational Behavior, 68(2), 347–367. 10.1016/j.jvb.2005.07.002

[apps12456-bib-0041] Kossek, E. E. , Ruderman, M. N. , Braddy, P. W. , & Hannum, K. M. (2012). Work–nonwork boundary management profiles: A person‐centered approach. Journal of Vocational Behavior, 81(1), 112–128. 10.1016/j.jvb.2012.04.003

[apps12456-bib-0042] Kubicek, B. , & Tement, S. (2016). Work intensification and the work‐home interface. Journal of Personnel Psychology, 15(2), 76–89. 10.1027/1866-5888/a000158

[apps12456-bib-0043] Lang, J. , & Kraus, T. (2012). Emotional vs. Cognitive rumination: Are they differentially associated with physical and psychological health? Proceedings of the 10th conference of the European Academy of Occupational Health Psychology.

[apps12456-bib-0044] Lazarus, R. S. , & Folkman, S. (1984). Stress, Appraisal, and Coping. Springer Publishing Company.

[apps12456-bib-0045] Lindner, P. , Frykheden, O. , Forsström, D. , Andersson, E. , Ljótsson, B. , Hedman, E. , Andersson, G. , & Carlbring, P. (2016). The Brunnsviken Brief Quality of Life Scale (BBQ): Development and psychometric evaluation. Cognitive Behaviour Therapy, 45(3), 182–195. 10.1080/16506073.2016.1143526 26886248 PMC4867878

[apps12456-bib-0046] Martin, L. , & Tesser, A. (1996). Some ruminative thoughts. In I. R. S. Wyer (Ed.). Ruminative thoughts: Advances in social cognition (pp. 1–47). Psychology Press.

[apps12456-bib-0047] Maslach, C. , Jackson, S. , & Leiter, M. P. (1996). Maslach Burnout Inventory (Third ed.). Consulting Psychologists Press.

[apps12456-bib-0048] Matthews, R. A. , Barnes‐Farrell, J. L. , & Bulger, C. A. (2010). Advancing measurement of work and family domain boundary characteristics. Journal of Vocational Behavior, 77(3), 447–460. 10.1016/j.jvb.2010.05.008

[apps12456-bib-0049] Matthews, R. A. , Kath, L. M. , & Barnes‐Farrell, J. L. (2010). A short, valid, predictive measure of work–family conflict: Item selection and scale validation. Journal of Occupational Health Psychology, 15(1), 75–90. 10.1037/a0017443 20063960

[apps12456-bib-0050] Michel, J. S. , Clark, M. A. , & Jaramillo, D. (2011). The role of the five factor model of personality in the perceptions of negative and positive forms of work–nonwork spillover: A meta‐analytic review. Journal of Vocational Behavior, 79(1), 191–203. 10.1016/j.jvb.2010.12.010

[apps12456-bib-0051] Nunnally, J. C. , & Bernstein, I. H. (1994). Psychometric theory. McGraw Hill.

[apps12456-bib-0052] Parent‐Thirion, A. , Biletta, I. , Cabrita, J. , Vargas, O. , Vermeylen, G. , Wilczyńska, A. , & Wilkens, M. (2016). Sixth European Working Conditions Survey—Overview Report (Eurofound). Publication Office of the European Union.

[apps12456-bib-0053] Podsakoff, P. M. , MacKenzie, S. B. , & Podsakoff, N. P. (2016). Recommendations for creating better concept definitions in the organizational, behavioral, and social sciences. Organizational Research Methods, 19(2), 159–203. 10.1177/1094428115624965

[apps12456-bib-0054] Powell, G. N. , & Greenhaus, J. H. (2010). Sex, gender, and the work‐to‐family interface: Exploring negative and positive interdependencies. Academy of Management Journal, 53(3), 513–534. 10.5465/amj.2010.51468647

[apps12456-bib-0055] Querstret, D. , & Cropley, M. (2012). Exploring the relationship between work‐related rumination, sleep quality, and work‐related fatigue. Journal of Occupational Health Psychology, 17(3), 341–353. 10.1037/a0028552 22746369

[apps12456-bib-0056] Rammstedt, B. , Danner, D. , Soto, C. J. , & John, O. P. (2020). Validation of the short and extra‐short forms of the Big Five Inventory‐2 (BFI‐2) and their German adaptations. European Journal of Psychological Assessment, 36(1), 149–161. 10.1027/1015-5759/a000481

[apps12456-bib-0057] Rosseel, Y. (2012). lavaan: An R package for structural equation modeling. Journal of Statistical Software, 48(2), 1–36. 10.18637/jss.v048.i02

[apps12456-bib-0058] Sauerland, M. (2018). Design your mind! Denkfallen entlarven und überwinden [Unmasking and overcoming thinking traps]. Springer. 10.1007/978-3-658-21462-3

[apps12456-bib-0059] Schaufeli, W. B. , Shimazu, A. , Hakanen, J. , Salanova, M. , & de Witte, H. (2019). An ultra‐short measure for work engagement. European Journal of Psychological Assessment, 35(4), 577–591. 10.1027/1015-5759/a000430

[apps12456-bib-0060] Schultze, M. (2018). Stuart: Subtests using algorithmic rummaging techniques. (R Package, Version 0.7.3). https://cran.r‐project.org/web/packages/stuart

[apps12456-bib-0061] Schultze, M. , & Eid, M. (2018). Identifying measurement invariant item sets in cross‐cultural settings using an automated item selection procedure. Methodology, 14(4), 177–188. 10.1027/1614-2241/a000155

[apps12456-bib-0062] Scott, M. J. , & Dryden, W. (1996). The cognitive‐behavioural paradigm. In Handbook of Counselling Psychology (pp. 156–179). Sage Publications, Inc.

[apps12456-bib-0063] Sedlar, N. , Šprah, L. , Tement, S. , & Sočan, G. (2015). Internal structure of an alternative measure of burnout: Study on the Slovenian adaptation of the Oldenburg Burnout Inventory (OLBI). Burnout Research, 2(1), 1–7. 10.1016/j.burn.2015.02.001

[apps12456-bib-0064] Shipp, A. J. , Edwards, J. R. , & Lambert, L. S. (2009). Conceptualization and measurement of temporal focus: The subjective experience of the past, present, and future. Organizational Behavior and Human Decision Processes, 110(1), 1–22. 10.1016/j.obhdp.2009.05.001

[apps12456-bib-0065] Sonnentag, S. (2018). The recovery paradox: Portraying the complex interplay between job stressors, lack of recovery, and poor well‐being. Research in Organizational Behavior, 38, 169–185. 10.1016/j.riob.2018.11.002

[apps12456-bib-0066] Sonnentag, S. , & Fritz, C. (2007). The Recovery Experience Questionnaire: Development and validation of a measure for assessing recuperation and unwinding from work. Journal of Occupational Health Psychology, 12(3), 204–221. 10.1037/1076-8998.12.3.204 17638488

[apps12456-bib-0067] Sonnentag, S. , & Fritz, C. (2015). Recovery from job stress: The stressor‐detachment model as an integrative framework. Journal of Organizational Behavior, 36(1), 72–103. 10.1002/job.1924

[apps12456-bib-0068] Soto, C. J. , & John, O. P. (2017). Short and extra‐short forms of the Big Five Inventory–2: The BFI‐2‐S and BFI‐2‐XS. Journal of Research in Personality, 68, 69–81. 10.1016/j.jrp.2017.02.004

[apps12456-bib-0069] Storbeck, J. , & Clore, G. L. (2007). On the interdependence of cognition and emotion. Cognition & Emotion, 21(6), 1212–1237. 10.1080/02699930701438020 18458789 PMC2366118

[apps12456-bib-0070] Tanzer, N. K. , & Sim, C. Q. E. (2013). Cross‐cultural validation. In K. D. Kenneth (Ed.), The encyclopedia of cross‐cultural psychology (pp. 278–286). 10.1002/9781118339893.wbeccp116

[apps12456-bib-0071] Tement, S. , Korunka, C. , & Pfifer, A. (2010). Toward the assessment of the work−family interface: Validation of the Slovenian versions of work−family conflict and work−family enrichment scales. Psihološka Obzorja, 19(3), 53–74.

[apps12456-bib-0072] Tement, S. , Zorjan, S. , Lavrič, M. , Poštuvan, V. , & Plohl, N. (2020). A randomized controlled trial to improve psychological detachment from work and well‐being among employees: A study protocol comparing online CBT‐based and mindfulness interventions. BMC Public Health, 20(1), 1708. 10.1186/s12889-020-09691-5 33198711 PMC7667737

[apps12456-bib-0073] Topp, C. W. , Østergaard, S. D. , Søndergaard, S. , & Bech, P. (2015). The WHO‐5 Well‐Being Index: A systematic review of the literature. Psychotherapy and Psychosomatics, 84(3), 167–176. 10.1159/000376585 25831962

[apps12456-bib-0074] van Katwyk, P. , Fox, S. , Spector, P. E. , & Kelloway, E. K. (2000). Using the Job‐Related Affective Well‐Being Scale (JAWS) to investigate affective responses to work stressors. Journal of Occupational Health Psychology, 5(2), 219–230. 10.1037/1076-8998.5.2.219 10784286

[apps12456-bib-0075] Vandenberg, R. J. , & Lance, C. E. (2000). A review and synthesis of the measurement invariance literature: Suggestions, practices, and recommendations for organizational research. Organizational Research Methods, 3(1), 4–70. 10.1177/109442810031002

[apps12456-bib-0076] Weigelt, O. , Gierer, P. , & Syrek, C. J. (2019). My mind is working overtime—Towards an integrative perspective of psychological detachment, work‐related rumination, and work reflection. International Journal of Environmental Research and Public Health, 16(16), 2987. 10.3390/ijerph16162987 31434205 PMC6719247

[apps12456-bib-0077] Wendsche, J. , de Bloom, J. , Syrek, C. , & Vahle‐Hinz, T. (2021). Always on, never done? How the mind recovers after a stressful workday? German Journal of Human Resource Management, 35(2), 117–151. 10.1177/23970022211004598

[apps12456-bib-0078] Wendsche, J. , & Lohmann‐Haislah, A. (2017). A meta‐analysis on antecedents and outcomes of detachment from work. Frontiers in Psychology, 7, 2072. 10.3389/fpsyg.2016.02072 28133454 PMC5233687

[apps12456-bib-0079] Wolff, H.‐G. , & Höge, T. (2011). Konflikte zwischen Arbeit und Familie [Conflicts between work and family]. Zeitschrift Für Arbeits‐ Und Organisationspsychologie, 55(3), 143–152. 10.1026/0932-4089/a000053

