# Supplementary material for: Development and validation of the Work–Home Integration Questionnaire (WHIQ)
Source: Appl Psychol. 2022 Dec 28;72(4):1694–734. doi: 10.1111/apps.12456 (PMC10952792; doi:10.1111/apps.12456)
Supplement: Supplementary file 1 — Table S1. The English Work‐Home Integration Questionnaire (WHIQ) Table S2. The German Work‐Home Integration Questionnaire (WHIQ) Table S3. The Slovene Work‐Home Integration Questionnaire (WHIQ) Table S4. Questionnaire Development of the Work‐Home Integration Questionnaire (WHIQ) Table S5. Confirmatory Factor Analysis and Invariance Test of the WHIQ in Study 2 Table S6. Confirmatory Factor Analysis of the WHIQ in Study 3 (T1) [file APPS-72-1694-s001.docx]

# Supplemental Material

## S.1. The Work-Home Integration Questionnaire (WHIQ)

Table 1. The English Work-Home Integration Questionnaire (WHIQ)

| *Please indicate how often, if at all, the involvements described below took place during the last year.* | | | | | |
| --- | --- | --- | --- | --- | --- |
| **During my leisure time …** | (Almost)  Never | Rarely | Sometimes | Often | (Almost)  Always |
| 1. … I worried about how I would deal with upcoming work tasks or issues. | 1 | 2 | 3 | 4 | 5 |
| 1. … I ruminated about things that went wrong at work. | 1 | 2 | 3 | 4 | 5 |
| 1. … I felt anxious because of upcoming work meetings or tasks. | 1 | 2 | 3 | 4 | 5 |
| 1. … I felt angry because of things that had happened at work. | 1 | 2 | 3 | 4 | 5 |
| 1. … I thought positively about upcoming work tasks. | 1 | 2 | 3 | 4 | 5 |
| 1. … I reflected on the things that went well at work. | 1 | 2 | 3 | 4 | 5 |
| 1. … I felt enthusiastic because of upcoming work tasks. | 1 | 2 | 3 | 4 | 5 |
| 1. … I felt proud because of my work-related achievements. | 1 | 2 | 3 | 4 | 5 |
| 1. … I responded to work-related phone calls or e-mails. | 1 | 2 | 3 | 4 | 5 |
| *a) Please rate the impact that this behavior has on your leisure time.* | *Negative -2 -1 0 1 2 Positive* | | | | |
| 1. … I studied work-related materials. | 1 | 2 | 3 | 4 | 5 |
| *a) Please rate the impact that this behavior has on your leisure time.* | *Negative -2 -1 0 1 2 Positive* | | | | |
| 1. … I talked about work-related things with my family or friends. | 1 | 2 | 3 | 4 | 5 |
| *a) Please rate the impact that this behavior has on your leisure time.* | *Negative -2 -1 0 1 2 Positive* | | | | |
| 1. … I organized work-related things with my colleagues or clients. | 1 | 2 | 3 | 4 | 5 |
| *a) Please rate the impact that this behavior has on your leisure time.* | *Negative -2 -1 0 1 2 Positive* | | | | |

Table 2. The German Work-Home Integration Questionnaire (WHIQ)

| *Bitte geben Sie an, wie oft, wenn überhaupt, die unten angeführten Aussagen im vergangenen Jahr aufgetreten sind.* | | | | | |
| --- | --- | --- | --- | --- | --- |
| **Während meiner Freizeit, …** | (Fast) Nie | Selten | Manch-mal | Oft | (Fast) Immer |
| 1. ... machte ich mir Sorgen darüber, wie ich die in der Arbeit anstehenden Aufgaben oder Probleme bewältigen würde. | 1 | 2 | 3 | 4 | 5 |
| 1. ... ging ich wiederholt in Gedanken die Dinge durch, die in der Arbeit schiefgegangen waren. | 1 | 2 | 3 | 4 | 5 |
| 1. ... war ich ängstlich aufgrund von anstehenden Meetings oder Aufgaben. | 1 | 2 | 3 | 4 | 5 |
| 1. ... ärgerte ich mich über Dinge, die in der Arbeit geschehen waren. | 1 | 2 | 3 | 4 | 5 |
| 1. ... dachte ich zuversichtlich über anstehende Arbeitsaufgaben nach. | 1 | 2 | 3 | 4 | 5 |
| 1. ... reflektierte ich die Dinge, die in der Arbeit gut gelaufen waren. | 1 | 2 | 3 | 4 | 5 |
| 1. ... war ich von den anstehenden Arbeitsanforderungen begeistert. | 1 | 2 | 3 | 4 | 5 |
| 1. ... war ich stolz auf meine Leistungen in der Arbeit. | 1 | 2 | 3 | 4 | 5 |
| 1. ... nahm ich arbeitsbezogene Anrufe an oder antwortete auf E‑Mails. | 1 | 2 | 3 | 4 | 5 |
| *a) Bitte bewerten Sie, welchen Einfluss dieses Verhalten auf Ihre Freizeit hat.* | *Negativ -2 -1 0 1 2 Positiv* | | | | |
| 1. ... ging ich arbeitsbezogene Unterlagen/Materialien durch. | 1 | 2 | 3 | 4 | 5 |
| *a) Bitte bewerten Sie, welchen Einfluss dieses Verhalten auf Ihre Freizeit hat.* | *Negativ -2 -1 0 1 2 Positiv* | | | | |
| 1. ... redete ich mit meiner Familie oder meinen FreundInnen über Arbeitsthemen. | 1 | 2 | 3 | 4 | 5 |
| *a) Bitte bewerten Sie, welchen Einfluss dieses Verhalten auf Ihre Freizeit hat.* | *Negativ -2 -1 0 1 2 Positiv* | | | | |
| 1. ... machte ich mit meinen KollegInnen oder KundInnen arbeitsbezogene Dinge aus. | 1 | 2 | 3 | 4 | 5 |
| *a) Bitte bewerten Sie, welchen Einfluss dieses Verhalten auf Ihre Freizeit hat.* | *Negativ -2 -1 0 1 2 Positiv* | | | | |

Table 3. The Slovene Work-Home Integration Questionnaire (WHIQ)

| *Prosimo, da označite, kako pogosto, če sploh, je v zadnjem letu prišlo do vpletenosti, ki je opisana spodaj.* | | | | | |
| --- | --- | --- | --- | --- | --- |
| **V prostem času …** | Nikoli | Redko | Včasih | Pogosto | Vedno |
| 1. ... me je skrbelo, kako se bom spoprijel/-a s prihajajočimi delovnimi nalogami ali problemi. | 1 | 2 | 3 | 4 | 5 |
| 1. ... sem kar naprej premleval/-a o stvareh, ki so šle pri delu narobe. | 1 | 2 | 3 | 4 | 5 |
| 1. ... sem se počutil/-a zaskrbljeno zaradi prihajajočih delovnih nalog ali sestankov. | 1 | 2 | 3 | 4 | 5 |
| 1. ... sem se počutil/-a jezno zaradi stvari, ki so se zgodile na delu. | 1 | 2 | 3 | 4 | 5 |
| 1. ... sem pozitivno razmišljal/-a o prihajajočih delovnih nalogah. | 1 | 2 | 3 | 4 | 5 |
| 1. ... sem premišljeval/-a o vseh stvareh, ki so se pri delu izšle dobro. | 1 | 2 | 3 | 4 | 5 |
| 1. ... sem se počutil/-a prijetno vznemirjeno zaradi prihajajočih delovnih nalog. | 1 | 2 | 3 | 4 | 5 |
| 1. ... sem se počutil/-a ponosno zaradi dosežkov pri delu. | 1 | 2 | 3 | 4 | 5 |
| 1. ... sem se odzival/-a na z delom povezane telefonske klice ali elektronsko pošto. | 1 | 2 | 3 | 4 | 5 |
| 1. *Prosimo ocenite vpliv tega dejanja na vaš prosti čas.* | *Negativen -2 -1 0 1 2 Pozitiven* | | | | |
| 1. ... sem proučeval/-a gradiva, ki so povezana z delom. | 1 | 1 | 1 | 1 | 1 |
| 1. *Prosimo ocenite vpliv tega dejanja na vaš prosti čas.* | *Negativen -2 -1 0 1 2 Pozitiven* | | | | |
| 1. ... sem s svojo družino ali prijatelji govoril/-a o stvareh, ki so povezane z delom. | 1 | 1 | 1 | 1 | 1 |
| 1. *Prosimo ocenite vpliv tega dejanja na vaš prosti čas.* | *Negativen -2 -1 0 1 2 Pozitiven* | | | | |
| 1. ...sem s svojimi sodelavci ali klienti organiziral/-a stvari, ki so povezane z delom. | 1 | 1 | 1 | 1 | 1 |
| 1. *Prosimo ocenite vpliv tega dejanja na vaš prosti čas.* | *Negativen -2 -1 0 1 2 Pozitiven* | | | | |

## S.2. Scoring of the Work-Home Integration Questionnaire (WHIQ)

Item numbers for scoring the WHIQ subscales are listed below:

1. Negative cognitive-affective involvement: 1, 2, 3, 4
2. Positive cognitive-affective involvement: 5, 6, 7, 8
3. Behavioral involvement: 9, 10, 11, 12

If participants indicate that they at least rarely (2=r*arely*, 3=*sometimes*, 4=*often*, 5=*[almost] always*) perform a specific WHI behavior, they are additionally asked to give a rating of that behavior on a scale from -2 (*very negative)* to +2 (*very positive)*.

The behavioral involvement valence total score is computed by multiplying each behavioral involvement item (9, 10, 11, 12; from which 1 is subtracted, so that the response format corresponds to 0=*never* and 4=*always*) with the respective valence item (9a, 10a, 11a, 12a; response format from -2=very negative to +2=very positive) and then calculating an overall mean. Participants who indicate that they *(almost) never* perform a specific WHI behavior (and who are thus not shown the corresponding valence item) receive a value of 0. The values of the behavioral involvement valence range from -8 to +8, with positive values for frequently occurring and positively perceived behavior and negative values for frequently occurring and negatively perceived behavior.

## S.3. Questionnaire Development of the Work-Home Integration Questionnaire (WHIQ)

Table 4. Questionnaire Development of the Work-Home Integration Questionnaire (WHIQ)

| **#** | **Original wording** | **Source** | **Original dimension** | | **Adapted version** | | **WHI Dimension** | | **Temporal orientation** | |
| --- | --- | --- | --- | --- | --- | --- | --- | --- | --- | --- |
| **1** | **I worried about how I would deal with a work task or issue** | **Flaxman et al. (2012)** | **Rumination** | | **During my leisure time, I worried about how I would deal with upcoming work tasks or issues.** | | **Negative cognitive-affective involvement** | | **Future** | |
| 2 | Do you think about tasks that need to be done at work the next day? | Cropley et al. (2012) | Problem-solving rumination | | During my leisure time, I ruminated about tasks that needed to be done at work the next day. | | Negative cognitive-affective involvement | | Future | |
| 3 | When you are at home, how often do you think about things you need to accomplish at work? | Carlson & Frone (2003) | Internal work-family interference | | During my leisure time, I worried about things that I needed to accomplish at work. | | Negative cognitive-affective involvement | | Future | |
| 4 | How often do you keep thinking about the negative things that happened at work even when you’re away from work? | Frone (2015) | Negative work rumination | | During my leisure time, I thought about the negative things that had happened at work. | | Negative cognitive-affective involvement | | Past | |
| 5 | My thoughts kept returning to a stressful situation at work | Flaxman et al. (2012) | Rumination | | During my leisure time, I thought back to stressful situations that happened at work. | | Negative cognitive-affective involvement | | Past | |
| **6** | **Before I start a task, I think long and hard about what could go wrong** | **Sauerland (2018)** | **Rumination** | | **During my leisure time, I ruminated about things what went wrong at work.** | | **Negative cognitive-affective involvement** | | **Past** | |
| 7 | How often do you replay negative work events in your mind even after you leave work? | Frone (2015) | Negative work rumination | | During my leisure time, I replayed negative work events in my mind. | | Negative cognitive-affective involvement | | Past | |
| 8 | During leisure time, I think about the negative sides of my work. | Binnewies et al. (2009) | Negative work reflection | | During my leisure time, I thought about the negative sides of my work. | | Negative cognitive-affective involvement | | - | |
| 9 | I mull over work issues during my time at home. | Capitano & Greenhaus (2018) | Permeability behavior | | During my leisure time, I mulled over work issues. | | Negative cognitive-affective involvement | | - | |
| 10 | Are you annoyed by thinking about work-related issues when not at work? | Cropley et al. (2012) | Affective rumination | | During my leisure time, I felt annoyed after an unsuccessful day at work. | | Negative cognitive-affective involvement | | Past | |
| **11** | **My job made me feel angry.** | **Van Katwyk et al. (2000)** | **Job-related affective well-being scale** | | **During my leisure time, I felt angry because of things that had happened at work.** | | **Negative cognitive-affective involvement** | | **Past** | |
| 12 | My job made me feel miserable. | Van Katwyk et al. (2000) | Job-related affective well-being scale | | During my leisure time, I felt bad because of mistakes I had made at work. | | Negative cognitive-affective involvement | | Past | |
| *13* |  | *Self-developed item* |  | | *During my leisure time, I felt guilty because of my unfinished work tasks.* | | *Negative cognitive-affective involvement* | | *Past* | |
| 14 | My job made me feel irritated. | Van Katwyk et al. (2000) | Job-related affective well-being scale | | During my leisure time, I felt irritated because of work-related issues. | | Negative cognitive-affective involvement | | - | |
| *15* | *My job made me feel confused.* | *Van Katwyk et al. (2000)* | *Job-related affective well-being scale* | | *During my leisure time, I felt confused because of work-related matters* | | *Negative cognitive-affective involvement* | | *-* | |
| **16** | **My job made me feel anxious.** | **Van Katwyk et al. (2000)** | **Job-related affective well-being scale** | | **During my leisure time, I felt anxious because of upcoming work meetings or tasks.** | | **Negative cognitive-affective involvement** | | **Future** | |
| 17 | My job made me feel frustrated. | Van Katwyk et al. (2000) | Job-related affective well-being scale | | During my leisure time, I felt frustrated by pending tasks at work. | | Negative cognitive-affective involvement | | Future | |
| 18 |  | Self-developed item |  | | During my leisure time, I felt overwhelmed because of upcoming work tasks. | | Negative cognitive-affective involvement | | Future | |
| **19** | **Do you think about tasks that need to be done at work the next day?** | **Cropley et al. (2012)** | **Problem-solving rumination** | | **During my leisure time, I thought positively about upcoming work tasks.** | | **Positive cognitive-affective involvement** | | **Future** | |
| 20 | After work I tend to think of how I can improve my work-related performance | Cropley et al. (2012) | Problem-solving rumination | | During my leisure time, I thought in a positive way about how I could improve my work-related performance. | | Positive cognitive-affective involvement | | Future | |
| 21 | I worried about how I would deal with a work task or issue | Flaxman et al. (2012) | Rumination | | During my leisure time, I thought positively about how I would accomplish upcoming work challenges. | | Positive cognitive-affective involvement | | Future | |
| 22 | How often do you think back to the good things that happened at work even when you’re away from work? | Frone (2015) | Positive work rumination | | During my leisure time, I thought back to good things that had happened at work. | | Positive cognitive-affective involvement | | Past | |
| 23 | How often do you replay positive work events in your mind even after you leave work? | Frone (2015) | Positive work rumination | | During my leisure time, I replayed positive work events in my mind. | | Positive cognitive-affective involvement | | Past | |
| **24** |  | **Self-developed item** |  | | **During my leisure time, I reflected on the things that went well at work.** | | **Positive cognitive-affective involvement** | | **Past** | |
| 25 | During leisure time, I think about the positive points of my job. | Binnewies et al. (2009) | Positive work reflection | | During my leisure time, I thought about the positive sides of my job. | | Positive cognitive-affective involvement | | - | |
| *26* | *During leisure time, I realize what I like about my job.* | *Binnewies et al. (2009)* | *Positive work reflection* | | *During my leisure time, I realized the upsides of my job.* | | *Positive cognitive-affective involvement* | | *-* | |
| 27 | You come home cheerfully after a successful day at work, positively affecting the atmosphere at home? | Geurts et al. (2005) | Positive work-home interaction | During my leisure time, I felt cheerful after a successful day at work. | | Positive cognitive-affective involvement | | Past | |  |
| **28** | **My job made me feel proud.** | **Van Katwyk et al. (2000)** | **Job-related affective well-being scale** | **During my leisure time, I felt proud because of my work-related achievements.** | | **Positive cognitive-affective involvement** | | **Past** | |  |
| 29 | My job made me feel happy. | Van Katwyk et al. (2000) | Job-related affective well-being scale | During my leisure time, I felt happy because of things that had happened at work. | | Positive cognitive-affective involvement | | Past | |  |
| 30 | My job made me feel inspired. | Van Katwyk et al. (2000) | Job-related affective well-being scale | During my leisure time, I felt inspired because of work-related things. | | Positive cognitive-affective involvement | | - | |  |
| 31 | My job made me feel satisfied. | Van Katwyk et al. (2000) | Job-related affective well-being scale | During my leisure time, I felt satisfied because of work-related things. | | Positive cognitive-affective involvement | | - | |  |
| *32* | *My job made me feel excited.* | *Van Katwyk et al. (2000)* | *Job-related affective well-being scale* | *During my leisure time, I felt excited about upcoming work meetings or tasks.* | | *Positive cognitive-affective involvement* | | *Future* | |  |
| *33* |  | *Self-developed item* |  | *During my leisure time, I was looking forward to pending challenges at work.* | | *Positive cognitive-affective involvement* | | *Future* | |  |
| **34** | **My job made me feel enthusiastic.** | **Van Katwyk et al. (2000)** | **Job-related affective well-being scale** | **During my leisure time, I felt enthusiastic because of upcoming work tasks.** | | **Positive cognitive-affective involvement** | | **Future** | |  |
| **35** | **I answer work-related correspondence (e.g., e-mail, texts, or phone calls) at home.** | **Capitano & Greenhaus (2018)** | **Permeability behavior** | **During my leisure time, I responded to work-related phone calls or e-mails.** | | **Behavioral involvement** | | **-** | |  |
| 36 |  | Self-developed item |  | During my leisure time, I checked work-related messages (e.g., via WhatsApp, Slack, Viber). | | Behavioral involvement | | - | |  |
| 37 |  | Self-developed item |  | During my leisure time, I read work-related articles/books or watched work-related videos/documentaries. | | Behavioral involvement | | - | |  |
| **38** | **It would be rare for me to read non-work-related materials at work.** | **Kossek et al. (2006)** | **Boundary management strategy measure** | **During my leisure time, I studied work-related materials.** | | **Behavioral involvement** | | **-** | |  |
| 39 | When you are at home, how often do you try to arrange, schedule, or perform job-related activities outside of your normal work hours? | Carlson & Frone (2003) | Internal work-family interference (IWIF) | During my leisure time, I performed work-related activities. | | Behavioral involvement | | - | |  |
| **40** | **I tend to not talk about work issues with my family.** | **Kossek et al. (2006)** | **Boundary management strategy measure** | **During my leisure time, I talked about work-related things with my family or friends.** | | **Behavioral involvement** | | **-** | |  |
| 41 |  | Self-developed item |  | During my leisure time, I discussed work-related things with my colleagues or clients. | | Behavioral involvement | | - | |  |
| **42** |  | **Self-developed item** |  | **During my leisure time, I organized work-related things with my colleagues or clients.** | | **Behavioral involvement** | | **-** | |  |

*Note.* Items in italic were dropped after the first pretest. Items in bold were considered in the final version.

## S.4. Confirmatory Factor Analysis and Measurement Invariance of the WHIQ in Study 2 and Study 3

Table 5. Confirmatory Factor Analysis and Invariance Test of the WHIQ in Study 2

| **Model** | **Sample** | **χ_SB_^2^** | ***df*** | ***p*** | **CFI** | **TLI** | **RMSEA  [90% CI]** | **SRMR** | **AIC** | **Model  Comparison** | **Δχ_SB_^2^** | **Δ*df*** | ***p*** | **ΔCFI** | **ΔRMSEA** |
| --- | --- | --- | --- | --- | --- | --- | --- | --- | --- | --- | --- | --- | --- | --- | --- |
| M1: 3-factor  model | a) Austrian | 112.51 | 51 | <.001 | .910 | .884 | .093 [.070-.116] | .087 | 4500.65 |  |  |  |  |  |  |
|  | b) Slovene | 169.36 | 51 | <.001 | .942 | .925 | .080 [.067-.094] | .065 | 12429.46 |  |  |  |  |  |  |
| M2: 2-factor model | a) Austrian | 282.68 | 53 | <.001 | .634 | .545 | .184 [.163-.205] | .151 | 4706.93 | M2a-M1a | 170.17 | 2 | <.001 | -.276 | .091 |
|  | b) Slovene | 718.33 | 53 | <.001 | .683 | .605 | .184 [.173-.197] | .181 | 13023.40 | M2b-M1b | 548.97 | 2 | <.001 | -.259 | .104 |
| M3: 2-factor model | a) Austrian | 205.97 | 53 | <.001 | .774 | .718 | .145 [.124-.166] | .112 | 4599.98 | M3a-M1a | 93.46 | 2 | <.001 | -.136 | .052 |
|  | b) Slovene | 462.71 | 53 | <.001 | .794 | .743 | .149 [.136-.161] | .137 | 12770.99 | M3b-M1b | 293.35 | 2 | <.001 | -.148 | .069 |
| M4: 2-factor model | a) Austrian | 227.30 | 53 | <.001 | .745 | .682 | .154 [.133-.175] | .124 | 4620.57 | M4a-M1a | 114.79 | 2 | <.001 | -.165 | .061 |
|  | b) Slovene | 619.55 | 53 | <.001 | .722 | .654 | .172 [.160-.185] | .192 | 12933.40 | M4b-M1b | 450.19 | 2 | <.001 | -.220 | .092 |
| M5: 1-factor  model | a) Austrian | 317.65 | 54 | <.001 | .552 | .452 | .202 [.181-.224] | .141 | 4771.02 | M5a-M1a | 205.14 | 3 | <.001 | -.358 | .109 |
|  | b) Slovene | 953.50 | 54 | <.001 | .553 | .454 | .217 [.205-.229] | .199 | 13322.67 | M5b-M1b | 784.14 | 3 | <.001 | -.389 | .137 |
| *Invariance Test* | |  |  |  |  |  |  |  |  |  |  |  |  |  |  |
| M1i: Configural model | | 283.11 | 102 | <.001 | .934 | .915 | .084 [.072-.096] | .066 | 16978.10 |  |  |  |  |  |  |
| M2i: Factor loadings constrained | | 332.01 | 111 | <.001 | .920 | .905 | .089 [.078-.100] | .079 | 17014.29 | M2i-M1i | 48.90 | 9 | <.001 | -.014 | .005 |
| M3i: Partial metric invariance | | 310.30 | 109 | <.001 | .927 | .912 | .085 [.074-.097] | .071 | 16992.24 | M3i-M1i | 27.19 | 7 | <.001 | -.007 | .001 |

*Note. N=*555 (*n* _Austrian_*=*150*, n* _Slovene_*=*405). M1: (1) negative cognitive-affective involvement, (2) positive cognitive-affective involvement, (3) behavioral involvement. M2: (1) negative cognitive-affective involvement and positive cognitive-affective involvement, (2) behavioral involvement. M3: (1) negative cognitive-affective involvement and behavioral involvement, (2) positive cognitive-affective involvement. M4: (1) positive cognitive-affective involvement and behavioral involvement, (2) negative cognitive-affective involvement. M5: (1) negative cognitive-affective involvement, positive cognitive-affective involvement, and behavioral involvement.

Table 6. Confirmatory Factor Analysis of the WHIQ in Study 3 (T1)

| **Model** | **χ_SB_^2^** | ***df*** | ***p*** | **CFI** | **TLI** | **RMSEA  [90% CI]** | **SRMR** | **AIC** | **Model  Comparison** | **Δχ_SB_^2^** | **Δ*df*** | ***p*** | **ΔCFI** | **ΔRMSEA** |
| --- | --- | --- | --- | --- | --- | --- | --- | --- | --- | --- | --- | --- | --- | --- |
| M1: 3-factor model | 121.97 | 51 | <.001 | .969 | .959 | .063 [.049-.078] | .060 | 10750.70 |  |  |  |  |  |  |
| M2: 2-factor model | 1155.19 | 53 | <.001 | .516 | .398 | .244 [.232-.256] | .222 | 11865.45 | M2-M1 | 1033.22 | 2 | <.001 | -.453 | .181 |
| M3: 2-factor model | 347.37 | 53 | <.001 | .868 | .835 | .128 [.115-.140] | .117 | 10998.93 | M3-M1 | 225.40 | 2 | <.001 | -.101 | .065 |
| M4: 2-factor model | 503.77 | 53 | <.001 | .793 | .742 | .160 [.147-.172] | .185 | 11185.37 | M4-M1 | 381.80 | 2 | <.001 | -.176 | .097 |
| M5: 1-factor model | 1324.28 | 54 | <.001 | .431 | .304 | 262 [.250-.274] | .229 | 12076.44 | M5-M1 | 1202.31 | 3 | <.001 | -.538 | .199 |

*Note.* *N=*379. M1: (1) negative cognitive-affective involvement, (2) positive cognitive-affective involvement, (3) behavioral involvement. M2: (1) negative cognitive-affective involvement and positive cognitive-affective involvement, (2) behavioral involvement. M3: (1) negative cognitive-affective involvement and behavioral involvement, (2) positive cognitive-affective involvement. M4: (1) positive cognitive-affective involvement and behavioral involvement, (2) negative cognitive-affective involvement. M5: (1) negative cognitive-affective involvement, positive cognitive-affective involvement, and behavioral involvement.
